# Gene duplication and relaxation from selective constraints of *GCYC* genes correlated with various floral symmetry patterns in Asiatic Gesneriaceae tribe Trichosporeae

**DOI:** 10.1371/journal.pone.0210054

**Published:** 2019-01-30

**Authors:** Kuan-Ting Hsin, Jing-Yi Lu, Michael Möller, Chun-Neng Wang

**Affiliations:** 1 Institute of Ecology and Evolutionary Biology, National Taiwan University, Taipei, Taiwan; 2 Department of Life Science, National Taiwan University, Taipei, Taiwan; 3 Royal Botanic Garden Edinburgh, Edinburgh, United Kingdom; University of Lausanne, SWITZERLAND

## Abstract

Floral bilateral symmetry is one of the most important acquisitions in flower shape evolution in angiosperms. Members of Gesneriaceae possess predominantly zygomorphic flowers yet natural reversal to actinomorphy have independently evolved multiple times. The development of floral bilateral symmetry relies greatly on the gene *CYCLOIDEA* (*CYC*). Our reconstructed *GCYC* phylogeny indicated at least five *GCYC* duplication events occurred over the evolutionary history of Gesneriaceae. However, the patterns of *GCYC* expression following the duplications and the role of natural selection on *GCYC* copies in relation to floral symmetry remained largely unstudied. The Asiatic tribe Trichosporeae contains most reversals to actinomorphy. We thus investigated shifts in *GCYC* gene expression among selected zygomorphic species (*Hemiboea bicornuta* and *Lysionotus pauciflorus*) and species with reversals to actinomorphy (*Conandron ramondioides*) by RT-PCR. In the actinomorphic *C*. *ramondioides*, none of the three copies of *GCYC* was found expressed in petals implying that the reversal was a loss-of-function event. On the other hand, both zygomorphic species retained one *GCYC1* copy that was expressed in the dorsal petals but each species utilized a different copy (*GCYC1C* for *H*. *bicornuta* and *GCYC1D* for *L*. *pauciflorus*). Together with previously published data, it appeared that *GCYC1C* and *GCYC1D* copies diversified their expression in a distinct species-specific pattern. To detect whether the selection signal (ω) changed before and after the duplication of *GCYC1* in Asiatic Trichosporeae, we reconstructed a *GCYC* phylogeny using maximum likelihood and Bayesian inference algorithms and examined selection signals using PAML. The PAML analysis detected relaxation from selection right after the *GCYC1* duplication (ω_pre-duplication_ = 0.2819, ω_post-duplication_ = 0.3985) among Asiatic Trichosporeae species. We propose that the selection relaxation after the *GCYC1* duplication created an "evolutionary window of flexibility" in which multiple copies were retained with randomly diverged roles for dorsal-specific expressions in association with floral symmetry changes.

## Introduction

Floral symmetry has long been thought to be one of the most extensively researched characters of trait evolution since the studies of Darwin in 1868 [1, 2, 3]. Among the various types of floral symmetry identified, zygomorphy and actinomorphy are the two major types across angiosperms. Zygomorphy, or bilateral symmetry, is characterized by a single plane of symmetry that separates flowers into two mirror images. By contrast, actinomorphy, or radial symmetry, is characterized by multiple planes of symmetry. Evolutionary reconstruction of floral symmetry states reveals that actinomorphy is the ancestral state yet multiple transitions to zygomorphy occurred across the angiosperm phylogeny [4, 5]. Zygomorphy is considered to be a key innovation, promoting specific ecological and evolutionary interactions between pollinators and floral shape [2, 3, 6, 7]. However, events of reversals from zygomorphy back to actinomorphy are also observed in several angiosperm lineages. Some of these reversions have been regarded as adaptations to rare pollinator and/or low pollination efficiency conditions such as high mountains or deep forests [8, 9].

Gesneriaceae is one of the families showing repeated shifts in floral symmetry in their evolutionary history. It is a family with over 3,500 species [10] in the order Lamiales with predominantly zygomorphic flowers, with more than half a dozen species showing actinomorphic flowers [10, 11, 12, 13, 14]. In an early classification Wang & al. [15] assigned the actinomorphic taxa *Ramonda* Rich., *Conandron* Siebold & Zucc, *Tengia* Chun, *Bournea* Oliv., *Thamnocharis* W.T. Wang, to a single tribe, Ramondeae, and considered this tribe also to be the most primitive in subfamily Cyrtandroideae (now Didymocarpoideae, see [10]), therefore, regarding actinomorphic taxa to represent a single ancestral lineage in Gesneriaceae. Burtt [16], based on the predominance of zygomorphic species and the scattered distribution of actinomorphic species in the taxonomic system, stated that taxa with actinomorphic flowers were probably reversal from the zygomorphic state and could not be regarded as ancestral. This was supported by molecular phylogenetic studies that indicated the family has a high number of species with reversals to actinomorphy [11, 13, 14, 17]. The most recent classification placed the five Old World actinomorphic genera, *Bournea*, *Conandron*, *Ramonda*, *Tengia*, *Thamnocharis* in the tribe Trichosporeae of subfamily Didymocarpoideae, and here in different subtribes: *R*. *myconii* (subtribe Ramondinae) and the other four in subtribe Didymocarpinae [10, 13, 14]. The subtribe Didymocarpinae includes the highest number of reversals to actinomorphy, with one event in *Conandron* [9], one in *Tengia* (now *Petrocodon*, see [18]), one in *Thamnocharis* (now *Oreocharis*, see [19]) and two in *Bournea* (now *Oreocharis*, see [19]) [13, 14, 17, 19]. The multiple reversals to actinomorphy in Trichosporeae make this tribe suitable for studying shifts of floral symmetry correlating to gene expression pattern between closely related zygomorphic and actinomorphic species. In addition, the actinomorphic flowers of species of two New World genera also evolved independently in different tribes within subfamily Gesnerioideae, *Bellonia* in tribe Gesnerieae and *Napeanthus* in tribe Napeantheae.

The genetics underlying floral symmetry was revealed by studies conducted on *CYCLOIDEA* (*CYC*) in *Antirrhinum majus* [20] in Plantaginaceae, a family in Lamiales closely related to Gesneriaceae [21]. In core Eudicots, *CYC* and all its homologues belong to the ECE-*CYC2* clade of the *CYC*/*TB1* subfamily in which the dorsal-specific expression evolved in their ancestor [22]. Extensive studies regarding the phylogeny and expression of ECE-*CYC2* genes among angiosperm lineages have demonstrated that multiple copies of *CYC2-*like genes from putative duplication events tend to be retained in both zygomorphic and actinomorphic lineages [22, 23, 24, 25, 26]. However, it is unclear why *CYC2-*like genes tend to retain multiple copies and whether these multiple *CYC2-*like copies exhibit diverse expression patterns in association with floral morphology and floral symmetry transitions.

Within Gesneriaceae, previous studies suggested at least four *GCYC* duplication events [23, 24] and these *GCYC* duplicates all belonged to ECE-*CYC2* clade [27, 28]. These duplications occurred at the subfamily to genus level. The first event occurred during the early diversification of the family, generating *GCYC1* and *GCYC2*. *GCYC2* is apparently lost in subfamily Gesnerioideae and perhaps Sanangoideae. Within tribe Trichosporeae, perhaps two additional independent *GCYC1* duplication events were identified. One duplication was detected across the tribe except for European species, forming two subclades, namely *GCYC1C* and *GCYC1D*, the other occurred solely for the African genus *Streptocarpus* [29] generating *GCYC1A* and *GCYC1B* [23, 27, 28, 30, 31, 32]. Together with the fact that Trichosporeae species have the highest number of reversals to actinomorphy, the high number of *GCYC1* duplications may imply that *GCYC* duplication correlates with the frequent floral symmetry transitions.

Gene duplication is generally required as precursor of functional divergence [33, 34, 35, 36, 37]. Model prediction and experimental data suggested that following gene duplication, the duplicated copies may have undergone functional divergence through neo-functionalization (shift to new gene expression domain) or sub-functionalization (modified expression domain partially overlapping with ancestral positions) or may have accumulated deleterious mutations and became non-functionalization (cease to express) [33, 36]. It has been proposed for *CYC* and other positively selected genes that after gene duplication, natural selection favors the fixation of mutations in one or more copies that adapt to divergent functions, and subsequently, sequential purifying selection acts to maintain new functions [34, 35, 36, 38, 39]. In other words, genetic constraints are temporarily lifted after duplication events (i.e. d_N_/d_S_ deviated from 0), and this relaxation provides the opportunity for duplicated genes to adopt divergent functions [37, 40]. For instance, in *Helianthus*, shifting strengths of positive selection (d_N_/d_S_ >1) have acted on three *CYC2-*like gene copies (*CYC2a*, *b*, and *c*) and functional divergence among them was inferred [41]. This was supported by their divergent expression patterns across the copies in which those of one clade were expressed in all floral tissues, another restricted to rays and disk florets, and the other limited to ray florets only, suggesting a case of sub- or neo-functionalization. In addition to functional divergence, loss of function in one copy, non-functionalization, is another possible fate for gene copies [33, 35, 36]. In wind-pollinated radially symmetrical flowers among six *Plantago* species, the loss of showy petals was correlated with a loss or non-expression of A-clade *CYC2*-like copies, a signature of non-functionalization [42]. Together, it is likely that natural selection can act on duplicated *CYC2-*like genes and subsequently their functions diverge, as inferred from shifts of their expression pattern and associated changes in flower morphology.

Only a few studies have examined the correlation between floral symmetry transition in Gesneriaceae and shifts in *GCYC* expression. Two studies were conducted on actinomorphic reversals in subtribe Didymocarpinae of tribe Trichosporeae, namely *Bournea leiophylla* (now *Oreocharis leiophylla*, see [19]) and *Tengia scopulorum* (now *Petrocodon scopulorum*, see [18]). Distinctive expression patterns between *GCYC* copies were detected in association with a transition to actinomorphy [31, 43]. In *O*. *leiophylla*, *BlCYC1* was found transiently expressed at the floral meristem initiation stage and then quickly vanished at later developmental stages. Thus, the loss of the ECE-*CYC2-*like gene expression in later flowering stage seems to correlate with the actinomorphy resulting in mature flowers [31]. In *P*. *scopulorum* and DA cultivar of African Violets, on the contrary, *GCYC* duplicated copies were detected across all petals and stamens, correlating with the development of dorsalized actinomorphy [32; 43]. These expression shifts of *GCYC* copies emphasize the possible associations of expression pattern change and resulting floral symmetry transition. Among zygomorphic Gesneriaceae species, such as *Chirita heterotricha* (now *Primulina heterotricha*, see [44]) and *Opithandra dinghushanensis* (now *Oreocharis dinghushanensis*, see [19]), it was found that the expression of duplicated *GCYC1* copies was mainly confined to the floral dorsal region, even though the expression pattern between copies usually differed and had expanded [27, 28, 45]. Overall, the expression pattern of *GCYC* duplications seemed to be correlated with their diversified expression patterns not only in actinomophic but also in zygomorphic species. This raises the possibility that potential expression pattern changes, thus functional divergence, has evolved frequently among *GCYC* duplicates. However, thus far, no integrated study has combined the evolutionary history, patterns of selective pressure, and expression patterns of *GCYC* copies to provide an overall interpretation to explain the reason why retention of multiple copies seemed to be preferred. Moreover, their association to floral symmetry remains to be studied.

The aim of this study was to investigate the patterns of gene expression and selective pressure of *GCYC* copies among species of tribe Trichosporeae in Gesneriaceae that contain species with zygomorphic and with actinomorphic flowers. In specific, we addressed (1) whether the expression patterns of these copies vary among zygomorphic and actinomorphy species, by studying two zygomorphic species *H*. *bicornuta* and *Lysionotus pauciflorus*, and one actinomorphic species *C*. *ramondioides*; and (2) whether the rate between nonsynonymous and synonymous substitutions (ω = d_N_/d_S_) between different copies changes after *GCYC* duplication events. Our results will reveal whether the expression pattern of *GCYC* varies from species to species in general, or whether copy-specific relaxation signals can be detected after gene duplication. The latter might suggest that evolutionary flexibility plays an important role in maintaining the diversity of flower morphology and floral symmetry transitions, underpinned by the retention of multiple *GCYC* duplicates in angiosperms.

## Materials and methods

### Ethics statement

Collection permission was granted by Yangmingshan National Park [Permission number: 20151155] for collecting plant species (*Hemiboea bicornuta* and *Lysionotus pauciflorus*).

### Species sampling and plant materials

In total, 57 Gesneriaceae species representing all major lineages, including 54 zygomorphic and 3 actinomorphic species (*Conandron ramondioides*, *Oreocharis leiophylla*, *Ramonda myconi*) covering two subfamilies Didymocarpoideae (Old World) and Gesnerioideae (New World) were selected to investigate the genealogy of *CYC-*like genes across Gesneriaceae (S1 Table). Forty-four *GCYC* copies from 27 species were isolated in this study by PCR with Gesneriaceae specific *GCYC* primers [46] (S1 Table). An additional 49 *GCYC* copies from 30 species were downloaded from GenBank, representing all publicly available *GCYC* sequences. A smaller dataset composing of 17 species of tribe Trichosporeae was used for analyses of the detection of selective pressure among *GCYC1C* and *GCYC1D* (excluding *GCYC1A* and *GCYC1B*) copies.

**Fig 1:**
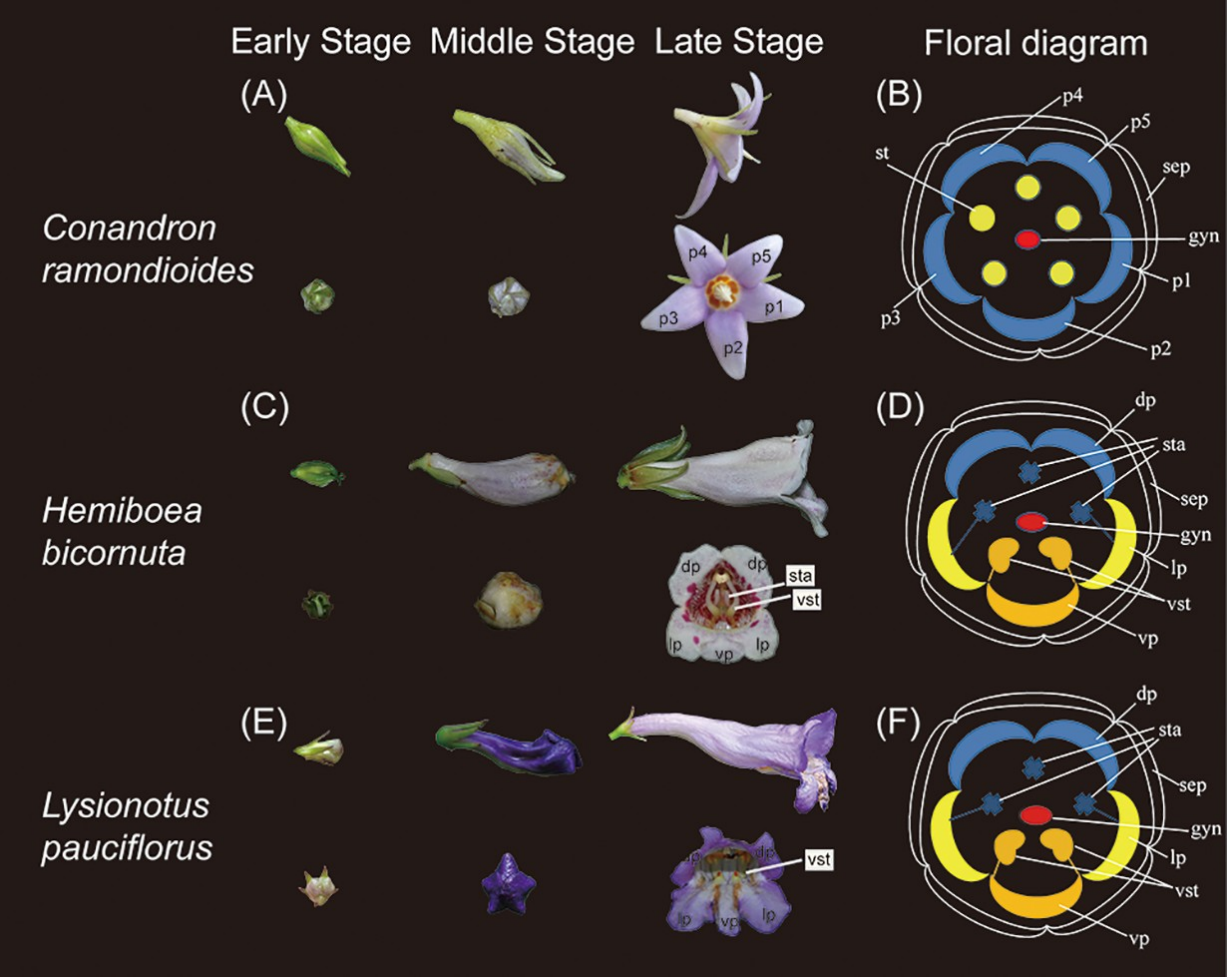
Flower morphology and developmental stages of *Conandron ramondioides*, *Hemiboea bicornuta*, and *Lysionotus pauciflorus*. (A) Three developmental stages (early, middle, and late) of *C*. *ramondioides—*Early Stage: petal length < sepal length; Middle Stage: petal length > sepal length; Late Stage: flower at anthesis. (B) Floral diagram: Five sepals (sep, black); five petals (from p1 to p5, blue); five stamens (st, yellow); gynoecium (gyn, red). (C) Three developmental stages of *H*. *bicornuta—*Early Stage: petal length < sepal length; Middle Stage: petal length > sepal size; Late Stage: flower at anthesis. (D) Floral diagram: sepals (sep, black); dorsal petals (dp, blue); lateral petals (lp, yellow); ventral petal (vp, orange); staminodes (sta, blue); ventral stamens (vst, orange); gynoecium (gyn, light blue). (E) Three development stages of *L*. *pauciflorus—*Early Stage: petal length < sepal length; Middle Stage: petal length > sepal size; Late Stage: flower at anthesis. (F) Floral diagram: sepals (sep, black); dorsal petals (dp, blue); lateral petals (lp, yellow); ventral petal (vp, orange); staminodes (sta, blue); ventral stamens (vst, orange); gynoecium (gyn, light blue).

To detect whether the *GCYC* expression pattern of target species showed chronological changes through developmental stages, flower buds of *C*. *ramondioides*, *H*. *bicornuta*, and *L*. *pauciflorus* were collected in the field and fixed in RNAlater (Ambion, USA) for locus-specific reverse transcription-polymerase chain reaction (RT-PCR). Flower buds of these three species were collected at three stages (see Fig 1): early stage (sepals closed with an erect and elongated peduncle), middle stage (sepals open, petal length longer than sepal length), and late stage (flower fully open with mature anthers). To locate organ specific expression of *GCYC*, early-stage floral buds were dissected. In this process, because of the floral organ differences between zygomorphic and actinomorphic species, we adopted two different processing steps. For the actinomorphic species, *C*. *ramondioides*, early-stage buds were dissected into seven regions: five single petals, stamens, and gynoecium. For the zygomorphic species, *H*. *bicornuta* and *L*. *pauciflorus*, early-stage buds were dissected into six categories: dorsal petals, lateral petals, ventral petal, stamens, staminodes, and gynoecium.

### Molecular cloning and sequence analysis

Total genomic DNA was extracted from fresh leaves or leaf material dried by silica gel following a CTAB protocol [47]. Partial sequences of *GCYC* of the 27 target species were amplified using a degenerate primer pair previously designed for *CYC2*-like genes in Gesneriaceae: *GCYCFs* and *GCYCR* [46]. Amplifications were performed on a DNA Programmable Thermal Cycler (ABI2700, USA) using the following conditions: initial denaturation at 94°C for 3 min; followed by 35 cycles at 94°C for 30 s, annealing at 55°C for 30 s, 72°C for 50 s; and a subsequent final extension at 72°C for 10 min. The PCR mixture (25 μL) contained 13 μL of premixture (Ampliqon, Denmark), 8.5 μL ddH_2_O, 1.25 μL of each primer, and 1μL of template DNA (20 ng). The PCR products were then cloned using the pGEM-T Easy Vector kit (Promega, USA), according to the manufacturer’s protocol. Plasmids containing the PCR products were further screened using colony PCR and purified with a Viogene kit (Taipei, Taiwan). For each PCR product, 8 clones were sequenced for checking the numbers of *GCYC* homologues. For sequencing, the T7 forward and reverse primers were used. All sequences were visually rechecked from the chromatograms. To evaluate the homology, sequences were aligned with all available *GCYC* sequences from GenBank and a phylogenetic tree reconstructed and *GCYC* clades and duplication events assigned (see below). All sequences were deposited in the National Center for Biotechnology Information (NCBI) nucleotide sequence database under the accession numbers provided in S1 Table.

### Locus-specific RT-PCR

Total RNA was extracted from the buds and dissected tissues of *H*. *bicornuta*, *L*. *pauciflorus*, and *C*. *ramondioides* using the Trizol (Invitrogen, USA) protocol. The quantity and quality of total RNA were determined using a Nanodrop Lite spectrophotometer (ThermoFisher Scientific, USA) and visualized by gel electrophoresis. Prior to cDNA synthesis, all RNA samples were treated with RQ1 DNase kit (Promega, USA) to eliminate possible genomic DNA contaminations. Each sample was checked for DNA contamination prior to RT-PCR (S2 Fig). Then, first-strand cDNAs were synthesized from total RNA with Superscript IV Rnase H–Reverse Transcriptase (Invitrogen, USA). The oligonucleotide sequences for primer pairs used to detect the expression pattern of target species are listed in supplementary file S1 Table. Expression levels of the *CYC*-like genes in floral buds and dissected floral parts were semi-quantified by comparing the intensity of the RT-PCR amplified bands on gels. RT-PCR products from each tissue were compared at 25, 30, 35 and 40 cycles to check for PCR saturation (S3 and S4 Figs). All products were examined via electrophoresis under a UV-light system (Omics Bio, Taipei, Taiwan). The RT-PCR conditions for amplifying the *GCYC* copies were as follows: denaturing at 94°C for 3 min, followed by 25–40 cycles (see above) of denaturation at 94°C for 30 s, annealing at gene specific settings (for *C*. *ramondioides CrCYC1C* and *CrCYC1D* at 53°C for 35 s; for *H*. *bicornuta HbCYC1C* and *HbCYC1D* at 55°C for 30 s; and for *L*. *pauciflorus LpCYC1C* and *LpCYC1D* at 53°C for 30 s), and extension at 72°C for 30 s, with a final extension step at 72°C for 10 min. 18S *rRNA* was used as an internal control to calibrate the amount of RNA in all RT-PCR reactions. All RT-PCRs were conducted with at least two biological replicates and two technical replicates for each developmental bud stage/floral organ to check the reproducibility of the results. The PCR products were examined under a UV-light system (see S3 and S4 Figs).

### Phylogeny reconstruction

The two homologs of *CYC2*-like genes of *Calceolaria arachnoidea*, *CaaCYC1* and *CaaCYC2*, were used as outgroup considering the close phylogenetic relationship of *C*. *arachnoidea* to Gesneriaceae [21, 24]. All *CYC*-like sequences were automatically aligned using MUSCLE [48] in MEGA v.6 [49], and the resulting alignment matrix visually refined based on amino acid translations (S1 File). Substitution model parameters were estimated in jModelTest2 [50]. The HKY+G model was selected as the best-fitting model based on the Bayesian Information criteria (BIC) [51]. Phylogenetic trees were reconstructed using Bayesian inference (BI) and maximum likelihood (ML) methods. Bayesian phylogenetic analyses were performed using MrBayes v.3.2.6 [52]. Two runs of four Markov chain Monte Carlo (MCMC) chains were run in parallel for 10 million generations, with a burn-in of 25% and sample frequency of every 1000^th^ generation. Convergence was assessed using the potential scale reduction factor (PSRF = 1.0) and the average standard deviation of split frequencies (<0.01). Additionally, the effective sampling size (ESS > 200) for all parameters and the traces of likelihoods were examined in Tracer v.1.6 [53]. A majority-rule consensus tree was reconstructed from the remaining trees and posterior probabilities determined in MrBayes using “sump” and “sumt” commands.

The ML phylogenetic analyses were performed using the PhyML 3.0 on-line web interface [54]. The HKY+G model derived from jModelTest2 was applied. Statistical support for nodes was assessed by an approximate likelihood ratio test (aLRT) [55] and conventional ML bootstrap algorithm (ML bootstrap) with 1000 replicates.

### Estimation of functional divergence of *GCYC* sequences between *1C* and *1D* clades

At the species level, coefficients of type I and II functional divergences (θ_I_ and θ_II_) were applied to assess selection on amino acid sites that are responsible for the functional divergence between duplicated *GCYC* clades, based on an ML approach implemented in DIVERGE v.3.0 [56]. Type I functional divergence aims to detect whether evolutionary rates vary between duplicate genes after gene duplication occurred, whereas type II functional divergence aims to detect the presence of radical changes in amino acid properties between duplicate genes (e.g. charge, hydrophobic, etc.) [57]. The type I and II methods calculate the parameter θ, where a θ value significantly greater than zero indicates functional divergence between the *GCYC1C* and *GCYC1D* clades.

### Detection of molecular evolutionary patterns of *GCYC* genes

*GCYC* sequences among clades at the species level were tested for detecting adaptive evolution after gene duplication by using the alternative and null branch model comparison of CODEML implemented in PAML v.4.0 [58]. A smaller subset including only *GCYC1* sequences of tribe Trichosporeae (excluding *GCYC1A* and *GCYC1B*) was used in the detection of selective pressure alterations after *GCYC1C*-*GCYC1D* duplication. For this, we realigned partial length (see S2 File) *GCYC1C* and *GCYC1D* sequences (including those of *CaaCYC1*, *RdCYC1*, *PsCYC1* and European *GCYC1*) and gaps were treated as missing data. A Bayesian phylogeny of this dataset was reconstructed based on the same program settings as above. To keep as many site information as possible, we set clean data = 0 in the PAML control file. The setting (clean data = 0) allows PAML to perform pairwise deletion of sites containing only missing data.

**Fig 2:**
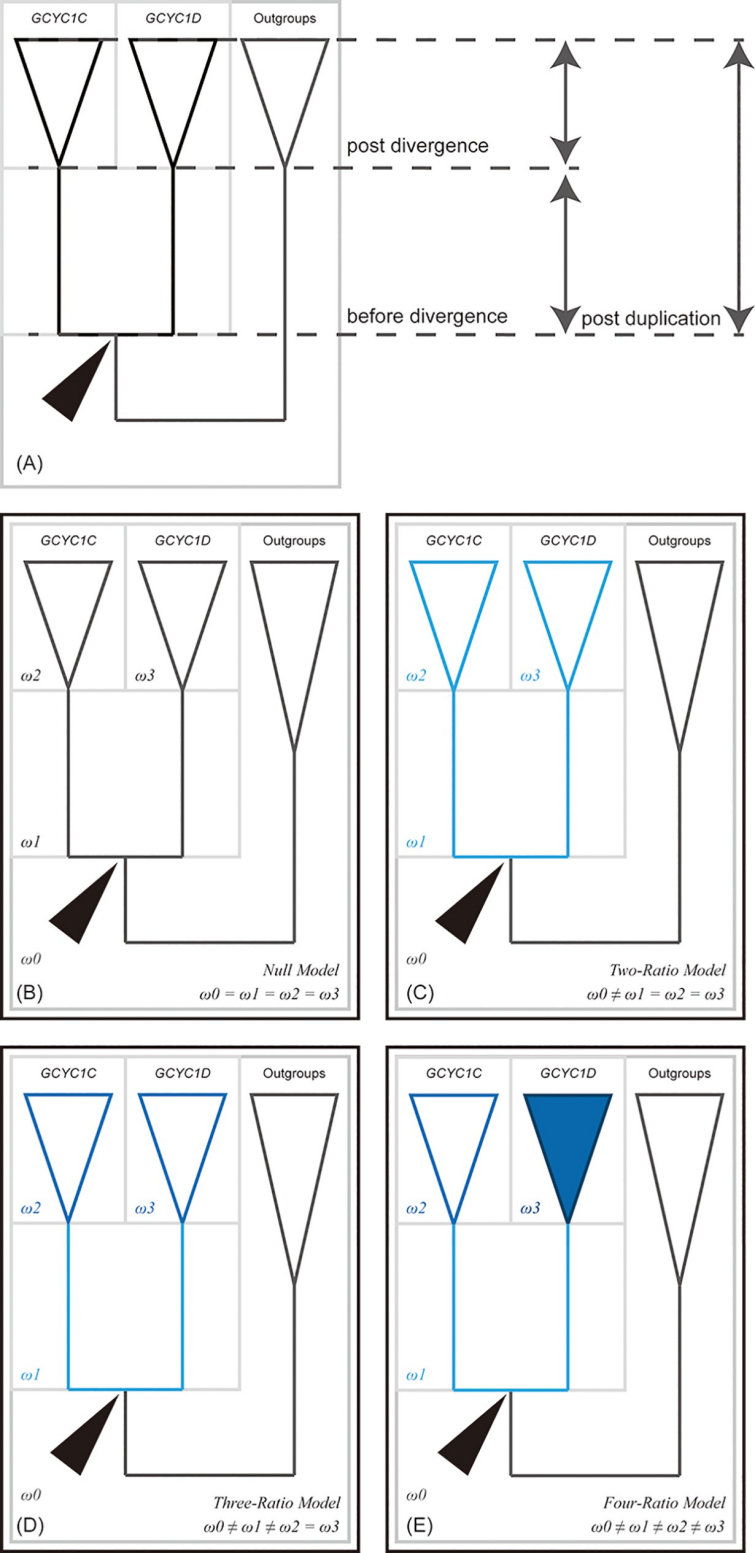
Branch models used in PAML analyses. (A) Schematic representation of the tree used in PAML. Arrowheads indicate the duplication event. Post duplication denotes all branches arising after the duplication event. Before divergence denotes the first branches following immediately the duplication event and post divergence denotes all subsequent branches. (B) One-ratio model, assumes equal ω across the tree. (C) Two-ratio model, assumes that ω changes pre- and post- duplication of *GCYC1C* and *GCYC1D*. Light blue line denotes same ω on branches post-duplication. (D) Three-ratio model, assumes ω varies before divergence (light blue lines), post divergence (dark blue lines) and outgroups (black lines). (E) Four-ratio model, assumes ω varies among branches before divergence (light blue lines), branches in *GCYC1C* clade (dark blue lines), branches in *GCYC1D* clade (dark blue triangle) and outgoups (black line).

To detect selection pressure changes along duplication events of *GCYC1C* and *GCYC1D*, we compared the ω value differences between pre-duplication, post-duplication but before divergence, and post divergence (Fig 2A). For model comparison, the null model (one-ratio model, Fig 2B) assumes a constant ω ratio for all *GCYC* branches, whereas the alternative model was constructed under the assumption that selective constraints changed following duplication events, similar to the strategy in [40]. In the modified model set, the first alternative model is a two-ratio model, which assumes two independent ω ratios: one ratio is before the duplication of *GCYC1C* and *GCYC1D* and the second ratio is after the duplication of *GCYC1C* and *GCYC1D* (Fig 2C). The second alternative model is a three-ratio model: the first ratio was assigned to all branches before the *GCYC1C* and *GCYC1D* duplication event, the second ratio was assigned to the two branches immediately after the duplication event, and the third ratio was assigned with equal values to all descendant branches after the divergence of *GCYC1C* and *GCYC1D* (Fig 2D). To test whether positive selection acted separately on *GCYC1C* or *GCYC1D* immediately after the duplication event, one additional three-ratio model was designed with the first ratio assigned to all branches before the duplication. But the second ratio was assigned to the branch leading to *GCYC1C* and all its descendant while the third ratio was assigned to *GCYC1D* and all its descendant branches. The four-ratio model separate the third ratio (along the divergence of *GCYC1C* and that of *GCYC1D*) in the three-ratio model into two clade-specific selective signals, namely *GCYC1C* or *GCYC1D* (Fig 2E). These models were tested against their inferior models (i.e. 4-ratio vs. 3-ratio) with 1 degree of freedom difference to examine the statistical support. Furthermore, in order to consider the selective pressure on specific sites or domains, branch-site models were used to increase the detectability and validate the results of branch models. Branch-site model A was built upon the assumption of a two-ratio branch model. The lineages before the duplication of *GCYC1C* and *GCYC1D* (second ratio) was assigned as foreground and the others as background (first ratio) to verify whether the ω value of foreground branches show relaxation (ω deviates from 0) or positive selection (ω greater than 1). This Branch-site model A was compared with a null model with fixed ω values of both foreground and background branches to 1 to assess any statistical support for positive selection [59]. Moreover, two separate branch-site model A with either *GCYC1C* or *GCYC1D* assigned as foreground were also applied in an attempt to discover hidden selective pressure on specific sites/domains in certain clades of these *GCYC* duplicates. Finally, clade model C, which allows more than one foreground clade, was used to examine whether ω values among foreground branches are different to those of background branches. Two models, one with post-duplication lineages as foreground and the other with *GCYC1C* and *GCYC1D* as different foregrounds, were used to examine the possibility of clade-specific selective pressures. The first clade model C (post-duplications as foreground) was tested against the null model m2a_rel [60] and the second clade model C (*GCYC1C* and *GCYC1D* as different foreground) was tested against the first clade model C. All alternative models were compared with their corresponding null models and tested using the likelihood ratio test (LRT) statistics [61].

## Results

### Evolutionary history of *GCYC* genes

**Fig 3:**
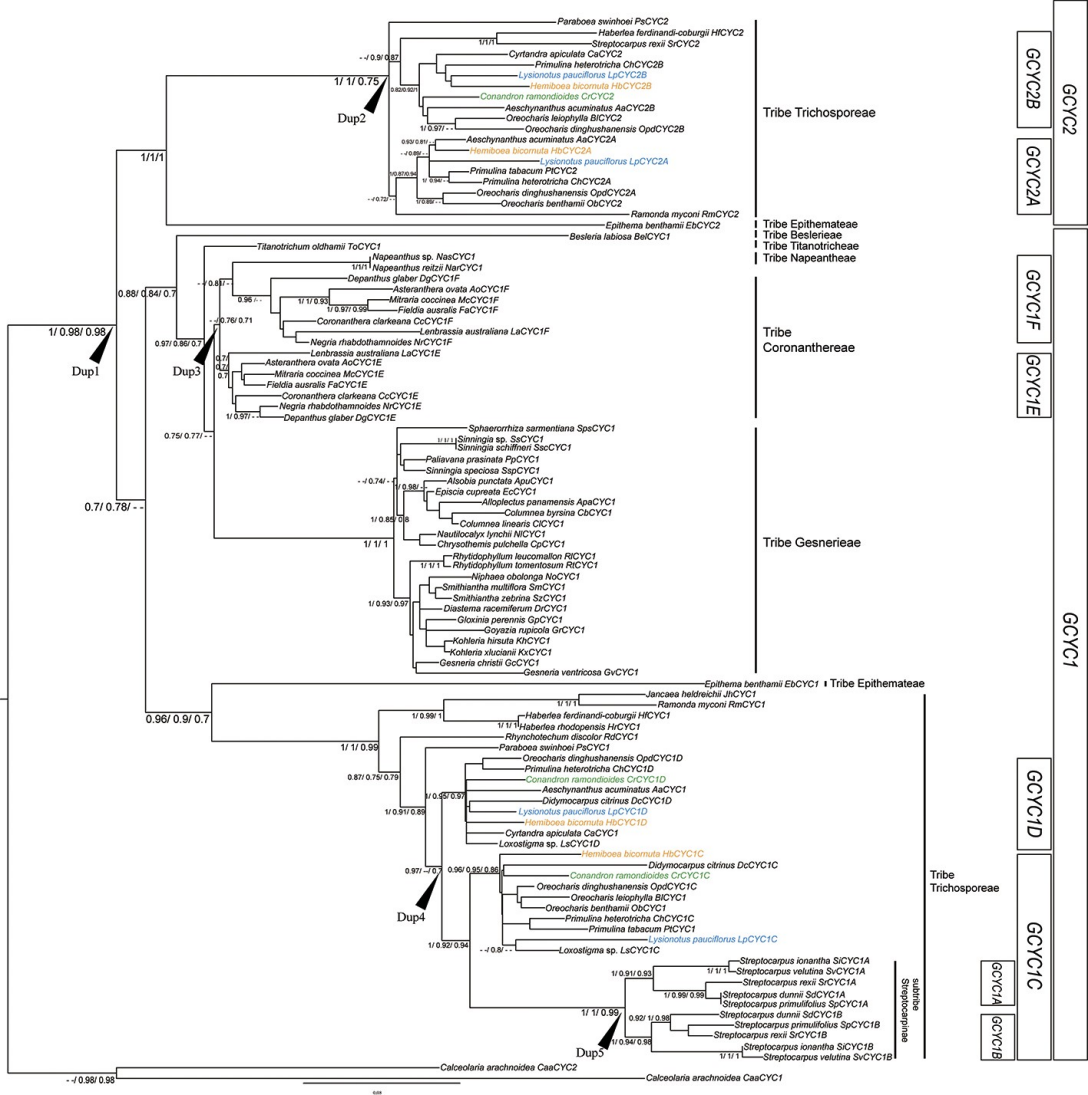
Genealogy and duplication events of *GCYC* in Gesneriaceae. Phylogram derived from a Maximum Likelihood (ML) analysis showing average branch lengths. Numbers along branches are BI, ML approximate likelihood value (aLRT), and ML bootstrap values. (--) indicates branch support value lower than 0.7 either in either BI, aLRT or ML bootstrap. *GCYC* copies of species used for expression studies are colored: *Conandron ramondioides* (light green); *Hemiboea bicornuta* (orange); *Lysionotus pauciflorus* (light blue). Dup1 to Dup5 indicate inferred duplication events.

In total, 98 homologs of 57 Gesneriaceae species were isolated in the present study or acquired from GenBank (S1 Table). The aligned sequence dataset was 840 bp in length. All *GCYC* genes contained partial conserved TCP, ECE, and R domains. Among the different copies, a *GCYC1D* specific 12 amino acid insertion located at about ten amino acid downstream the R domain was also found (see S1 Fig) The numbers of copies varied among the clades in the phylogenetic tree across the family (Fig 3). All subfamily Gesnerioideae (New World) species had only one copy, namely *GCYC1*. For several subfamily Didymocarpoideae species, at least two copies of *GCYC* were identified, *GCYC1* and *GCYC2*.

To infer duplication events of *GCYC* genes, we reconstructed the evolutionary history of *GCYC* genes using both Bayesian Inference (BI) and Maximum Likelihood (ML) algorithms. Only nodes with BI or ML probability value over 0.7 (BI > 0.7 or aLRT > 0.7 or ML bootstrap > 0.7) were regarded as a reliable clade. According to our criteria, five duplication events can be inferred across the phylogeny (Fig 3). The first duplication appeared at the basal part of the tree and gave rise to two well-supported clades (Fig 3, Dup1; BI: 1, aLRT: 0.98, ML bootstrap: 0.98): *GCYC1* (BI: 0.7, aLRT: 0.78) and *GCYC2* (BI: 1, aLRT: 1, ML bootstrap: 0.75). Most of the subfamily Didymocarpoideae species (Old World) had both *GCYC1* and *GCYC2* copies but all subfamily Gesnerioideae species (New World) retained only *GCYC1* copies. From the tree topology in Fig 3, the split between *GCYC1* and *GCYC2* was an ancestral duplication in the common ancestor of Gesneriaceae, yet Gesnerioideae species appeared to have lost their *GCYC2* copy or it has not yet been found. Within *GCYC2*, there was one further duplication (Fig 3, Dup2; BI: 1, aLRT: 1, ML bootstrap: 0.75) perhaps occurring in the common ancestor of Old World tribe Trichosporeae species as species of several subtribes across the tribe contained both *GCYC2A* and *GCYC2B*. Within *GCYC1*, there were three further independent lineage-specific duplication events. One duplication (Fig 3, Dup3; aLRT: 0.76, ML bootstrap: 0.71) perhaps occurred in the common ancestor of New World tribes Coronanthereae and Napeantheae thus *GCYC1* in these species further duplicated into *GCYC1E* (BI: 0.7, aLRT: 0.7, ML bootstrap: 0.7) and *GCYC1F* (aLRT: 0.81). Among Old World tribe Trichosporeae species, a second *GCYC1* duplication event (Fig 3, Dup4; BI: 0.97, ML bootstrap: 0.7) gave rise to *GCYC1C* (BI: 1, aLRT: 0.92, ML bootstrap: 0.94) and *GCYC1D* (BI: 1, aLRT: 0.95, ML bootstrap: 0.97) and seemed to occur in the common ancestor of the Asiatic and Malesian subtribe Didymocarpinae species and African and Madagascan subtribe Streptocarpinae species, since species of subtribes Ramondinae, Leptoboeinae and Loxocarpinae species possessed only one *GCYC1* copy (Fig 3). The African and Madagascan species of subtribe Streptocarpinae seemed to have lost their *GCYC1D* copy. On the other hand, within subtribe Streptocarpinae species a further gene duplication event had occurred (Fig 3, Dup5; BI: 1, aLRT: 1, ML bootstrap: 0.99), resulting in *GCYC1A* (BI: 1, aLRT: 0.91, ML bootstrap: 0.93) and *GCYC1B* (BI: 1, aLRT: 0.94, ML bootstrap: 0.98).

### Expression pattern and variation among *GCYC* copies

**Fig 4:**
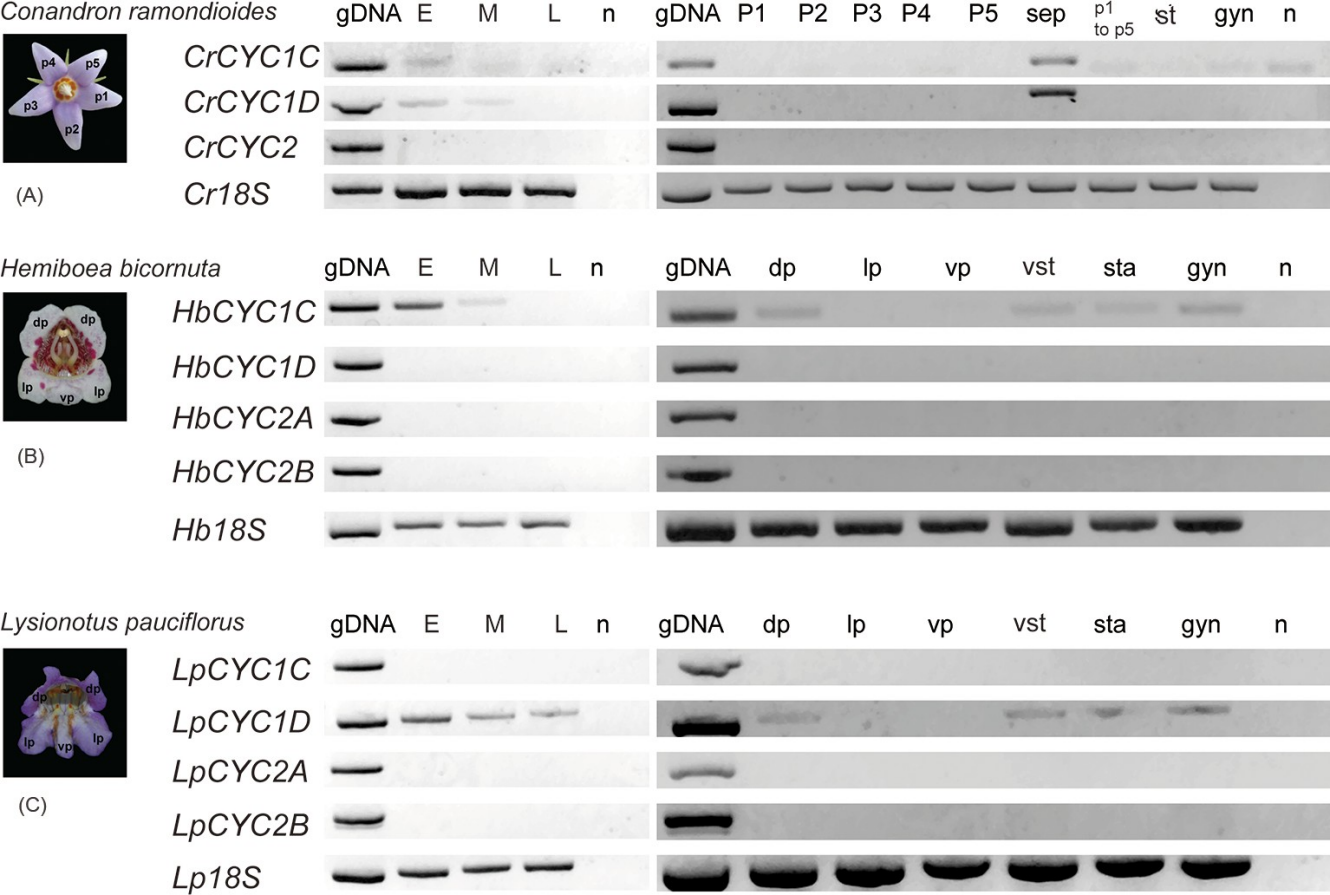
Expression pattern of *GCYC* in floral parts of *Conandron ramondioides*, *Hemiboea bicornuta*, and *Lysionotus pauciflorus*. (A) *Conandron ramondioides*. (B) *Hemiboea bicornuta*. (C) *Lysionotus pauciflorus*. E: Early Stage flower bud, M: Middle Stage flower bud, L: flower bud at anthesis; n denotes no-template control; dp: dorsal petals, lp: lateral petals vp: ventral petal; p1 to p5: pooled *C*. *ramondioides* petals; st: pooled stamens (*C*. *ramondioides*); vst: ventral stamens, sta: staminodes (*H*. *bicornuta*, *L*. *pauciflorus*); gyn: gynoecium; sep: pooled sepals (*C*. *ramondioides*). A very faint band appears sometimes in no-template controls in *C*. *ramondioides* and represent primer dimer artefacts (see S5 Fig).

The expression patterns of *GCYC* copies varied from species to species (Fig 4). For the actinomorphic *C*. *ramondioides*, three copies namely *CrCYC1C*, *CrCYC1D* and *CrCYC2* were identified. Both *CrCYC1C* and *CrCYC1D* were detected during the early (E) and mid (M) flower developmental stages (Fig 4). In dissected floral organs, both *CrCYC1C* and *CrCYC1D* were not detected in single petals (P1, P2, P3, P4, P5), pooled petals (P1 to P5), pooled stamens (st), or the gynoecium, but were detected in pooled sepals (sep). *CrCYC2* was not detected throughout the flower developmental stages. This implies that the actinomorphy of *C*. *ramondioides* perhaps resulted from the entire loss of *GCYC* expression in the petal whorl. In the zygomorphic *H*. *bicornuta*, four copies of *GCYC* were isolated, namely *HbCYC1C*, *HbCYC1D*, *HbCYC2A* and *HbCYC2B* (Fig 3). Only *HbCYC1C* was found expressed in early flower buds (E), whereas *HbCYC1D* was not expressed in flowers (Fig 4). Organ specific expression of *HbCYC1C* was detected in dorsal petals (dp), staminodes (sta) which are situated in the dorsal part of the flower, ventral stamens (vst), and gynoecium (gyn). For the zygomorphic *L*. *pauciflorus*, also four copies of *GCYC* were isolated, namely *LpCYC1C*, *LpCYC1D*, *LpCYC2A* and *LpCYC2B* (Fig 3). In contrast to *Hemiboea*, *LpCYC1D*, instead of *LpCYC1C*, was expressed in all floral bud stages in *L*. *pauciflorus* (Fig 4). Similarly, *LpCYC1D* had an organ specific expression in the dorsal petals (dp), staminodes (sta), stamens (st), and the gynoecium (gyn). *GCYC2* was also not expressed in both zygomorphic species. Dorsal petal and staminode specific expression of *GCYC* was detected in both zygomorphic species. But in *H*. *bicornuta*, *GCYC1C* (*HbCYC1C*) was the more prominently expressed copy while in *L*. *pauciflorus*, *GCYC1D* (*LpCYC1D*) was the more strongly expressed copy.

### Patterns of molecular evolution in Trichosporeae *GCYC1C and GCYC1D* sequences

**Fig 5:**
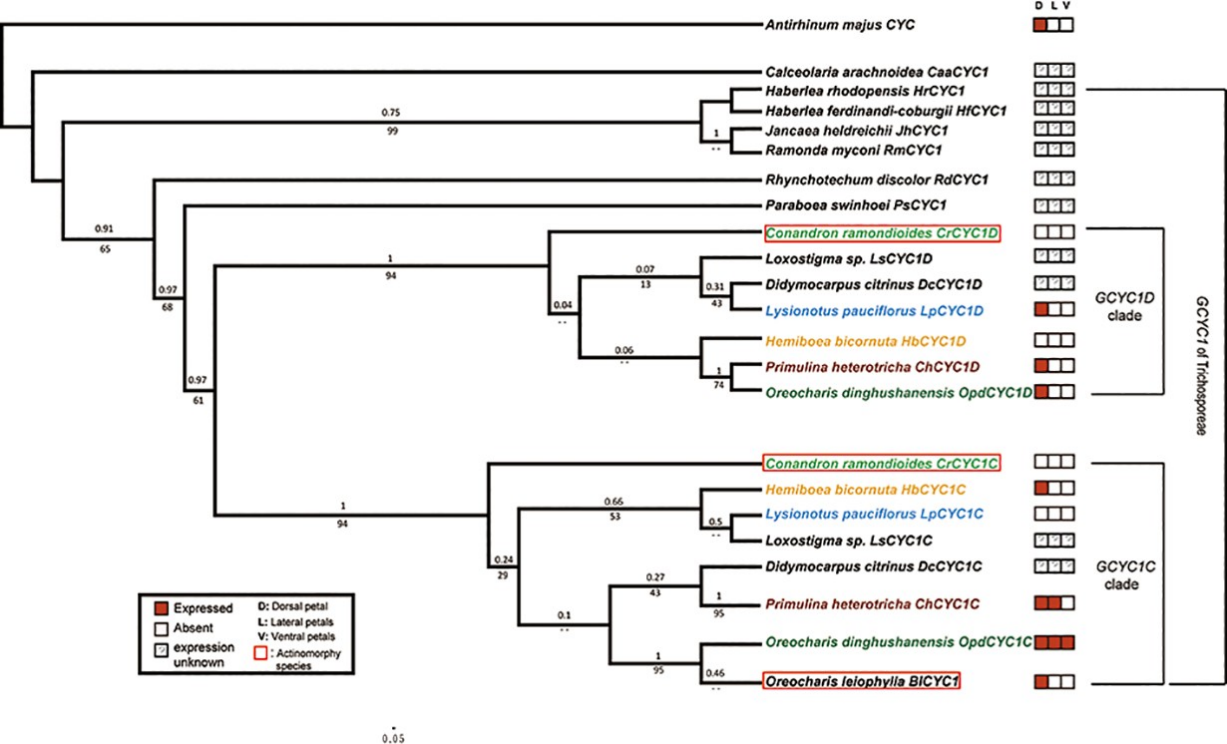
*GCYC1* phylogenetic tree with summarized *GCYC1C* and *GCYC1D* expression pattern in tribe Trichosporeae species. BI cladogram based on *GCYC1* sequences of species in tribe Trichosporeae. Numbers above branches and below branches are approximate likelihood and posterior probability values obtaining from ML and BI analyses respectively. *GCYC1* expression patterns are shown in color by organ: D: dorsal petal, L: lateral petal, V: ventral petal; red squares represent presence and white squares absence of gene expression in the respective flower part; square with ‘?’ denote unknown expression. Expression pattern were compiled from this work and previous studies ([27, 28, 31]). *GCYC* genes isolated from actinomorphy species in boxes. Light green font: *Conandron ramondioides*, dark green font: *Oreocharis dinghushanensis*, light blue font: *Lysionotus pauciflorus*, Orange font: *Hemiboea bicornuta*, Brown font: *Primulina heterotricha*, Black font: *Oreocharis leiophylla*.

The smaller *GCYC* sequence subset used for detecting patterns of selection was 744 bp in length and had 383 variable sites. The BI and ML tree topologies were congruent but fully resolved in comparison to the large analysis (Figs 3 and 5). Our two-ratio branch model of the selection analysis revealed that ω significantly increased from before duplication (pre-duplication) (ω_0_ = 0.2819) to duplication into *GCYC1C* and *GCYC1D* (ω_post-duplication_ = 0.3985*) (Model A1, Table 1, Fig 2C), suggesting a relaxation from genetic constraints. This was further confirmed by the results for the clade model C (Model C1, Table 1), which detected a relaxation signal of selective pressure after the *GCYC1C*/*1D* duplication (post-duplication) (ω_0_ = 0.0469, ω_post-duplication_ = 0.1968*, *p*-value <0.05). Furthermore, the three-ratio branch model (Model A2-1, Table 1) was statistically supported to be superior to the two-ratio model (Model A1), suggesting that a selection pressure was maintained on both *GCYC1C* and *GCYC1D* lineages right after duplication (ω_before divergence_ = 0.1344), but eventually relaxed after divergence of the *GCYC1C*/*1D* lineages (ω_after divergence_ = 0.431*, Table 1, Fig 2D). The four-ratio branch model (Model A3, Table 1) compared if selective pressures were different between *GCYC1C* and *GCYC1D*, but its likelihood was not significantly better than the three ratio model (Model A2-1, Table 1, Fig 2E), indicating the selective pressure was the same in *GCYC1C* and *GCYC1D*. Similarly, the clade model C2 (Table 1), which assigned *GCYC1C* and *GCYC1D* as separated foregrounds, was not different from the null model C0, therefore failing to detect *GCYC1C*/*1D* clade-specific positive selection changes. These results suggested that the relaxation signal observed after *GCYC1C*/*1D* duplication was most likely caused by relaxation rather than a positive selection difference acting on either *GCYC1C* or *GCYC1D* after their divergence. However, branch site model A, the attempts to detect whether certain sequence regions are under selection, failed to find any site or specific lineage subject to natural selection or relax from selection (S3 Table). Attempts to uncover Type I and Type II functional divergences between *GCYC1C* and *GCYC1D* with DIVERGE however, failed to find certain sites which underwent functional divergence (S4 Table).

**Table 1:** Parameter estimates under branch and branch-site models of *GCYC1C* and *GCYC1D* in tribe Trichosporeae.

Model (foreground branches)	Log Likelihood (lnL)	LRT (20δL)^a^ statistics (Models; df; p value^b^)	ω estimates (proportion)
**Branch models**			
One-ratio model			
A0: Null model (constant ω)	-4235.3261		ω = 0.3438
Two-ratio model			
A1: (Post-duplication)	-4233.28989	4.07 (A1 vs. A0; 1; 0.04*)	ω_0_ = 0.2819; **ω**_**post-duplication**_ **=** 0.3985
		Three-ratio model			
A2-1: (before divergence, post divergence)	-4229.9248	6.73 (A2-1 vs. A1; 1; 0.009**)	ω_0_ = 0.2834; ω_before divergence_ = 0.1344; **ω**_**post divergence**_ **=** 0.4310
		A2-2: (*GCYC1C*, *GCYC1D*)	-4233.2526	0.0744 (A2-2 vs. A1; 1; 0.785)	ω_0_ = 0.2819; ω_*GCYC1C*_ = 0.3875; ω_*GCYC1D*_ = 0.4122
Four-ratio model					
A3: (Post-duplication, *GCYC1C*, *GCYC1D*)	-4229.8949		
		0.06 (A3 vs. A2-1; 1; 0.806)	ω_0_ = 0.2834; ω_post-duplication_ = 0.1344; ω_*GCYC1C*_ = 0.4198; ω_*GCYC1D*_ = 0.4446
		**Clade model C**			
C0: M2a_rel (null model)	-4193.8004		ω_P0_ = 0.4308 (P_0_ = 0.5075), ω_P1_ = 1 (P_1_ = 0.1518) ω_P2_ = 0.0594 (P_2_ = 0.3408)
	C1: (Post-duplication)	-4189.2893	9.022 (C1 vs. C0; 1; 0.002**)	ω_0_ = 0.5244; ω_Post-duplication_ = 0.5244 (P_0_ = 0.3755), ω_0_ = ω_Post-duplication_ = 1 (P_1_ = 0.1335) ω_0_ = 0.0469; **ω**_**Post-duplication**_ **=** 0.1968 (P_2_ = 0.4911)
C2: (*GCYC1C*, *GCYC1D*	-4188.9258	0.7271 (C2 vs. C0; 2; 0.39)	ω_0_ = ω_*GCYC1C*_ = ω_*GCYC1D*_ = 0.4985 (P_0_ = 0.3953), ω_0_ = ω_*GCYC1C*_ = ω_*GCYC1D*_ = 1 (P_1_ = 0.1408) ω_0_ = 0.0469; ω_*GCYC1C*_ = 0.1529; ω_*GCYC1D*_ = 0.2285 (P_2_ = 0.4911)

^a^ The likelihood ratio test (LRT) was used to test the statistical differences between models by calculating the value of double the difference between likelihood values (2δL).

^b^ *p*-values were calculated by comparing the statistics 2δL with the *X*^*2*^ distribution (degree of freedom equals to the difference of number of parameters between models).

*, <5% significance level

**, <1% significance level. Significant *p*-value highlighted in bold.

## Discussion

### Lineage specific duplications and losses of *GCYC* in Gesneriaceae

With extensive samplings of 96 *GCYC* sequences from 57 representative Gesneriaceae species, we reconstructed a comprehensive *GCYC* phylogeny which allowed the inference of at least five duplication events and two gene loss events across the family during the evolution history of *GCYC*. The monophyly of *GCYC1* and *GCYC2* across the Gesneriaceae confirmed hypotheses from previous studies that the two paralogs originated in the most recent common ancestor of Gesneriaceae after its split from Calceolariaceae and subsequently experienced lineage-specific duplications within Gesneriaceae [23, 24]. This *GCYC1/2* duplication was first identified from a small set of Gesneriaceae species [23] that suggested that *GCYC* duplications may be involved in the evolution of floral symmetry. Consistent with previous studies, only *GCYC1* is present in the New World species [24, 62]. It is interesting to note that *GCYC2* appears to have been immediately lost in the New World clade (i.e. subfamily Gesnerioideae) after the initial *GCYC* duplication. Lineage-wide losses appear to have played a significant part in the *GCYC* evolution in Gesneriaceae as indicated by the loss of *GCYC1D* for subtribe Streptocarpinae (Fig 3; [23]).

Within the *GCYC1* clade, the independent duplications in the Old World tribe Trichosporeae (*GCYC1C*/*1D*) and New World tribe Coronanthereae (*GCYC1E/ 1F*) implied they arose from different mechanisms. It is quite likely that a whole genome duplication was involved in the duplication of *GCYC* in Coronanthereae since cytological data revealed that many of the species in this tribe are polyploid [63, 64]. In tribe Trichosporeae, however, no such mechanism can be invoked since the duplication into *GCYC1C*/*1D* at the tribe level and *GCYC1C* into *GCYC1A*/*1B* duplication at subtribal level species with similar chromosome numbers are involved [63, 64]. It is as yet unclear why there are more duplications of *GCYC* events occurring particularly in Trichosporeae species, but retaining multiple *CYC* copies would probably allow these to acquire diversified function as discussed below.

### Association between expression pattern shifts and floral symmetry after *GCYC* duplications

Shifts of gene expression patterns among duplicated copies are usually an implication for functional divergence. Frequent duplications of ECE-*CYC2* together with the evolution of diverse expression patterns among these copies have played a major role in their functional divergence [65]. In *A*. *majus* [25] and other Eudicots species, the process of frequent *CYC* duplications and subsequent functional divergences upon selection can be summarized into three stages: 1) duplication; 2) relaxed from purifying selection immediately after the duplication, this allowed both duplicated copies have equal activities (e.g. same expression levels) as their ancestral gene; and 3) their excess cumulative activities enables one copy to become less constraint than its original function by drift, thus facilitating the accumulation of more deleterious mutations (3a. non-functionalization), or shift into a new expression pattern (3b. neo-functionalization), or both copies could retain the ancestral function by gradually reducing their excess activities/expressions to be cumulatively equal to the level of the ancestral copy (3c. functionally redundancy), or one copy partially overlap its expression/activity to the other with complementary roles (3d, sub-functionalization, one major/one helper). For example, the duplication of *CYC* in *A*. *majus* and the stronger phenotype of *cyc* relative to *dich* mutants suggest that they diverged into one major (i.e. *CYC*) / the other helper (i.e. *DICH*) function on floral morphology. This is also evident from their expression level differences in that both *CYC* and *DICH* were found to be expressed in the adaxial flower region, but *CYC* showed a broader and stronger expression on the dorsal side than *DICH* [20, 66, 67].

The duplication of *CYC2-*like genes among zygomorphic species in Dipsacales has resulted in a shift in expression out of the ventral part of the corolla (dorsal and lateral lobes) of both duplicates, *DipsCYC2B* and *DipsCYC2A* [68]. But in derived species, *DipsCYC2A* became limited in expression to just two dorsal lobes while *DipsCYC2B* maintained its expression in the dorsal and lateral lobes. In species with actinomorphic reversals, both *CYC2-*like copies shifted to expand their expression to either lateral lobes (*DipsCYC2A*, dorsal + lateral) or further to the ventral lobe (*DipsCYC2B*, dorsal + lateral + ventral). This supports the view that duplications may promote shifts of gene expression patterns among duplicates. Because *DipsCYC2A* and *DipsCYC2B* partly overlap in their expression to each other in Dipsacales species, this consequence of the duplication can be viewed as a sub-functionalization process.

The loss of association between plant and oil bee pollinator was found repeatedly in several unrelated Malpighiaceae lineages, and in each case these loss was associated with changes in floral symmetry (corresponding to shifts of *CYC2A* expression) and the loss of *CYC2B* expression in flower buds. This may indicate non-functionalization of one of the duplicates, *CYC2B*, helps to alter flower morphology [69, 70]. The rise of heterogeneous gene expression patterns after gene duplication events is therefore a common feature for the evolution of *CYC2-*like genes in Eudicot species. In the present study, the expression pattern shifts of duplicated *GCYC1* copies became even more diversified compared to these above examples (see below).

### *GCYC1* duplications generate diversified expression patterns correlating with floral symmetry in a species-specific manner

**Fig 6:**
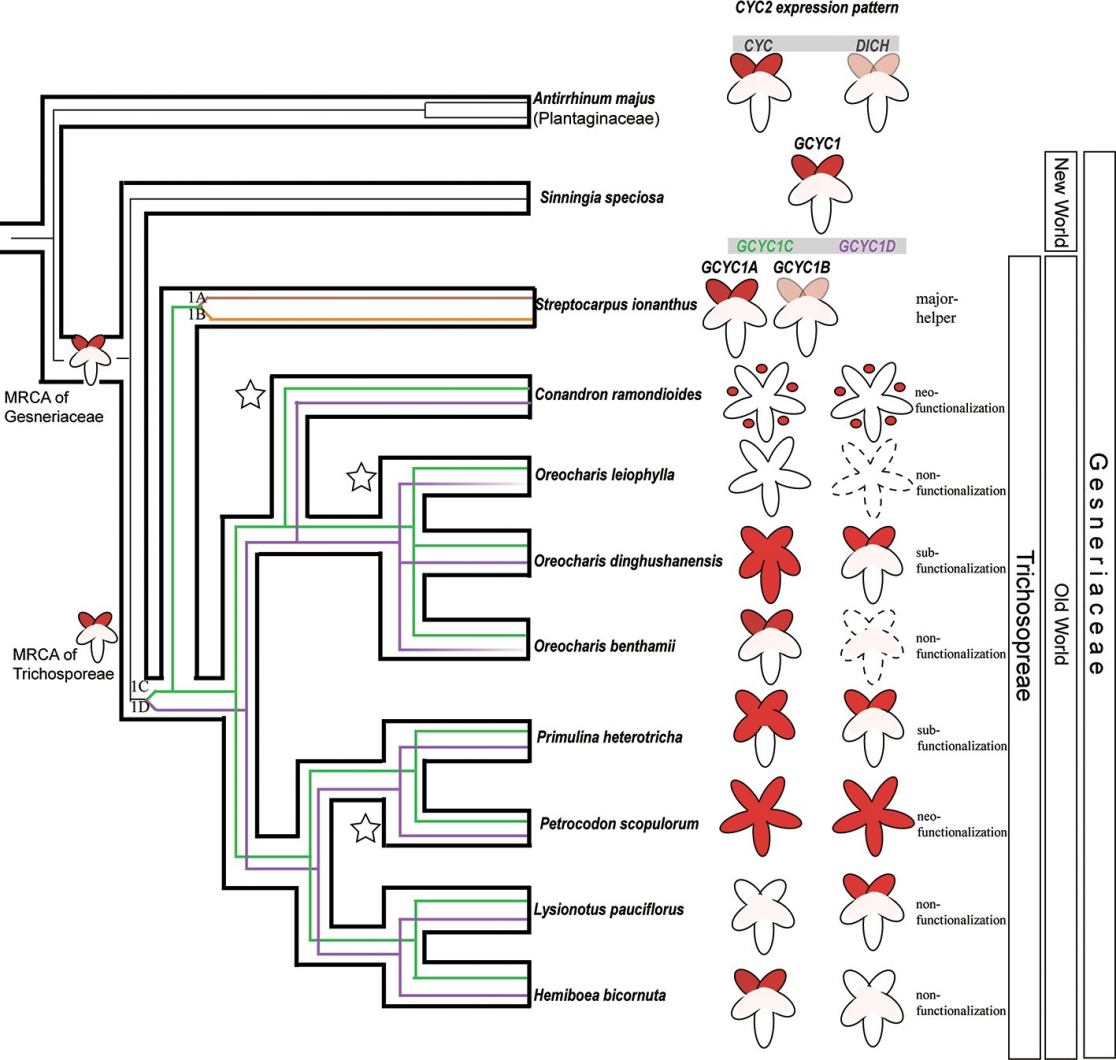
Reconstructed ECE*-CYC2* evolution emphasizing shifts of *GCYC* expression pattern after gene duplication in correlation to floral symmetry transitions among selected Asiatic Trichosporeae species.

The expression of *GCYC1C* and *GCYC1D* copies post duplication seemed to exhibit a species-specific expression pattern among Trichosporeae species as observed from our data and previous results (Fig 5, Fig 6). As summarized in Fig 6, the duplication of *GCYC1* into *GCYC1C* and *GCYC1D*, as well as *GCYC1C* duplicated into *GCYC1A* and *GCYC1B*, resulted in diversified expression patterns among these copies in Trichosporeae species and they varied species by species.

The hollow-branch phylogeny indicates the phylogenetic relationships among selected Trichosporeae species based on [13]. The superimposed solid-line phylogenies depict *GCYC* genealogy and duplication events. Black lines: ancestral ECE-*CYC2* lineages in the outgroup *Antirrhinum majus* and the New World representing sister species, *Sinningia speciosa*. *GCYC1* paralogs originated in the common ancestor of New World and Old World Gesneriaceae species. Because *CYC*/*DICH* are dorsal petal specifically expressed, it is hypothesized that *GCYC1* expression at the most recent common ancestor (MRCA) of Gesneriaceae is also dorsal specific. After *GCYC1* duplication into *GCYC1C* (green lines) and *GCYC1D* (purple lines) in the MRCA of Trichosporeae species, their expression patterns greatly diversified and differentiated into “species-specific” expression patterns (see discussion). *GCYC1C* underwent one further duplication among the genus *Streptocarpus* species into *GCYC1A* (brown line) and *GCYC1B* (orange line) [23]. Stars denote the three independently evolved reversals to actinomorphic lineages. The corolla (petal) cartoons indicate expression of ECE-*CYC2* including *GCYC1* copies. Red color indicates expression domains of *GCYC1* copies within petals. Pink indicates a relatively weak expression of *DICH* and *GCYC1B*, the helper copy for *CYC* in *Antirrhinum majus* and that for *GCYC1B* in *Streptocarpus ionanthus*, respectively. The dotted outlines indicate the potential loss of expression of a certain gene copy.

Because in the sister clade of tribe Trichosporeae, such as the New World tribe Gesnerieae represented by *Sinningia speciosa*, *GCYC1* is dorsally expressed, as in *Antirrhinum majus*, *GCYC1* expression in the MRCA of Trichosporeae is probably dorsal specific (Fig 6). However, upon the duplication of *GCYC1* into *GCYC1C* and *GCYC1D* copies, their expression patterns became much diversified but these expression shifts correlated to their floral symmetry forms. Among three independently evolved actinomorphic species (braches marked with star in Fig 6), in *C*. *ramondioides*, both *CrCYC1C* and *CrCYC1D* were found to have completely lost their expression in all five petals (Fig 4). But both *CrCYC1C* and *CrCYC1D* were still expressed in floral buds, particularly in the sepals. Thus, the reversal to actinomorphy in *C*. *ramondioides* is not a *CYC* loss of function mutation, instead, it is due to the expression shifts of *GCYC1* away from the petal whorl into sepals. This shifted expression pattern suggests that *CrCYC1C* and *CrCYC1D* had acquired a neo-functionalization for flower development due to an expressions shift to sepals (Fig 6). In another reversal to actinomorphy case, *Petrocodon scopulorum* (*Tengia*), however, both *GCYC1C* and *GCYC1D* are ubiquitously expressed in all five petals to result in a dorsalized actinomorphic flower [43]. This expression pattern shifts from the ancestrally dorsal-specific pattern can also be regarded as neo-functionalization, yet it was independently evolved in different lineage to *C*. *ramondioides* (Fig 6). In one other actinomorphic reversal case, *Oreocharis leiophylla* (*Bournea*), *GCYC1C* (*BlCYC1*) although transiently expressed in the dorsal region of the flower, the expression did not persist to later stages and thus the flower developed radially symmetry [31]. The other duplicated copy *GCYC1D* was likely lost in *O*. *leiophylla* because PCR was failed to amplify this copy even primers designed from conserved domain were used [31]. Non-functionalization of *GCYC1C* and *GCYC1D* is a plausible consequence after their duplication from *GCYC1*.

For zygomorphic species, as revealed in this study, non-functionalization of randomly selected *GCYC1C* or *GCYC1D* paralogs was found, such as in *Lysionotus pauciflorus* (loss of *GCYC1C* expression but retaining *GCYC1D* dorsal expression) and *Hemiboea bicornuta* (loss of *GCYC1D* expression but retaining *GCYC1C* instead) (Figs 5 and 6). A similar randomly selected non-functionalization case can be inferred from one other zygomorphic lineage such as *Oreocharis benthamii* where *GCYC1C* expression is retained dorsally but *GCYC1D* expression is likely lost (Fig 6) [30]. Therefore, the recruitment of dorsal specific expression of *GCYC1C* or *GCYC1D* is likely not predetermined among Trichosporeae species, rather, these copies are randomly fixed from species to species.

Expression shifts inferring sub-functionalization (expression domains of one duplicated copy expanded/reduced but still overlapping with the other copy with ancestral expression pattern) among *GCYC1* duplicates can also be inferred from two other zygomorphic Trichosporeae lineages. In *Primulina heterotricha*, *GCYC1C* (*ChCYC1C*) expression was found extended to the lateral petals from dorsal petals, while *GCYC1D* (*ChCYC1D*) expression remained restricted to the dorsal petals (Figs 5 and 6) [28]. While in *Oreocharis dinghushanensis*, *GCYC1C* (*OpdCYC1C*) expression further extended to all petals but *GCYC1D* (*OpdCYC1D*) remained expressed in the dorsal petals only (Fig 6) [27]. In *Saintpaulia ionanthus* (now *Streptocarpus ionanthus*, see [29]), one additional duplication of *GCYC1C* resulted in one major copy, *GCYC1A*, strongly expressed in dorsal petals, and the other copy, *GCYC1B*, weakly expressed there [32] (Fig 6). Similar to the case for *CYC* and *DICH*, this *GCYC1A* / *GCYC1B* duplication indicated a functionally redundant duplication in which perhaps one major and one helper function evolved. Alternatively, in the absence of empirical functional data, *GCYC1B* may be on a trajectory to non-functionalization. This is the evolutionarily the most recent duplication (Fig 3) and copy *GCYC1B* maybe at present redundant and may eventually become lost, or attains a new function.

It is clear that multiple *GCYC1* duplications (*GCYC1C* / *GCYC1D* and *GCYC1A* / *GCYC1B*) and their resulting diversified expression patterns correlate well with zygomorphic and actinomorphic development in Trichosporeae species, and these patterns evolved randomly in different copies in a “species-specific” manner.

### Relaxation signals detected after duplication of *GCYC1C* and *GCYC1D*

Given the fact that *GCYC1* duplications in the Trichosporeae species have evolved species-specific gene expression pattern, we investigated whether differential selective pressures were apparent among the *GCYC* copies (Table 1, Fig 2). The divergence of both *GCYC1C* and *GCYC1D* copies show a signature of relaxation from purifying selection among the *GCYC1* duplicates. Furthermore, the post duplication relaxation signal persisted throughout their divergence. Our two-ratio branch and clade C model results (two-ratio model) both revealed a relaxation signal immediately after the *GCYC1* duplication (post-duplication) (model A1: *ω*_0_ = 0.2819, *ω*_post-duplication_ = 0.3985*, model C1: *ω*_0_ = 0.0469, *ω*_post-duplication_ = 0.1968*, Table 1, Fig 2c). However, there was no indication of positive selection acting specifically on either the *GCYC1C* or *GCYC1D* clade (model A3 and C2, no statistic support for natural selection on *GCYC1C* or *GCYC1D* as foreground, Table 1). This seems to suggest that the duplication of *GCYC1* into *GCYC1C* or *GCYC1D* resulted in relaxation of both copies among Asiatic Trichosporeae species with one copy randomly shifting into a new expression pattern (see Fig 6). However, our results for the three-ratio branch model further indicated that the relaxation signal was still detectable throughout the divergence of these *GCYC1C* and *GCYC1D* copies after the duplication (model A2-1: *ω*_0_ = 0.2834, *ω*_post divergence_ = 0.4310*, Table 1, Fig 2D), and its likelihood was significantly better than for the two-ratio model above. This implies that the *GCYC1* relaxation signal was not only maintained immediately after the duplication but also throughout the divergence of *GCYC1C* and *GCYC1D*, a phenomenon similar to the balancing selection whereby all duplicated copies tended to be retained because no selective constraint acted on a specific copy. In this way, their functions were randomly fixed in each species, as revealed by their species-specific expression pattern.

This species-specific *GCYC1C* and *GCYC1D* expression pattern among Trichosporeae species following *GCYC1* duplication is therefore unique within Gesneriaceae. A similar conclusion was reached for Ranunculaceae zygomorphic species (tribe Delphinieae species) in which all *CYC2*-like paralogs were expressed in a species-specific pattern in correlation to their distinct perianth architecture [38]. But the selection analysis failed to detect any positive selection and relaxation from purifying selection signals among the copies, except for one duplicate, *RANACYL1a*, that experienced a higher level of functional constraint for maintaining zygomorphy (*ω* value close to 0) [38].

Extensive duplications of *CYC2*-like genes have been reported for species in Lamiales, to which Gesneriaceae belong to, Eudicots and monocot species. However, very few studies investigated what selective regime favored the retention of multiple copies post duplication, and different conclusions were drawn. In Plantaginaceae tribe Antirrhineae, immediately after *CYC*/*DICH* duplication, relaxation from purifying selection was detected only in the functional domain along the lineage leading to *DICH*, but not for the *CYC* lineage [25]. However, we found that both *GCYC1C* and *GCYC1D* copies experienced relaxation post duplication. Perhaps due to this finding the expression patterns of both *GCYC1C* and *GCYC1D* copies were able to diversify resulting in the species-specific patterns found here.

Several studies focused on the retention of *CYC2*-like copies in a range of plants such as Zingiberales, sunflower (Asteraceae) and *Lupinus densiflorus* (Fabaceae), and reported that one paralog post duplication experienced positive selection or had deviated expression patterns thus allowing for functional divergence [41, 71, 72]. In fact, a “selection mosaic” hypothesis, in which episodic positive selection, purifying selection and relaxed purifying selection have been suggested to act parallel or in tandem to account for the high rates of duplication and diversification in *CYC2*-like genes [71]. Recent findings on the evolution of *CYC2*-like genes from Lamiales and *Anacyclus* (Asteracaeae) provide support for this view [39, 65]. Our results of persistent relaxation after duplications of *GCYC1* copies, however, adds a new facet to explain why *CYC2*-like genes are retained in multiple copies.

On the other hand, we cannot rule out the possibility that the relaxation signal was actually resulting from a mixture of global and copy specific selection signals. Our conclusions here are preliminary and could be substantiated by adding more samples from Trichosporeae species, although it is unlikely that this would result in less variation in *GCYC* expression. In fact, in *CYC2*-like duplication-selection analysis among species of Lamiales, Zhong and Kellog [65] did not find any relaxation or positive selection signal among Gesneriaceae *CYC2*-like copies because of their limited sampling size. The reason why our analysis, on the contrary, found continuous relaxation signals among *GCYC1* duplicated copies, is probably due to the more extensive species sampling.

One possible explanation for the retaining of multiple *GCYC* copies is to generate diverse flower morphology that may have resulted from a functional-morphological adaptation for Trichosporeae species to cope with potential pollinator shifts [8, 9, 69, 70]. Positive selection on *CYC2*-like copies has been found associated with larger lateral petals (wings) and narrower dorsal petals in *Lupinus densiflorus*, that play a putative role to attract pollinators for outcrossing [71]. Furthermore, maintaining multiple *GCYC1* copies is also a prerequisite for evolutionary flexibility (see below).

### Potential role of evolutionary flexibility since the duplication of *GCYC1*

Duplicated genes provide opportunities to acquire evolutionary flexibility and can be recruited for divergent functions, such as *Hox* genes in animals [73]. Traditionally, the fate of duplicated genes was believed to be consistent within single duplicates by either functional divergence or random fixation at the copy level [40, 74]. We argue that *GCYC1* also acquired this flexibility similar to the concept of a toolbox of genes for generating diverse functions. By plotting the available expression data of Trichosporeae to the *GCYC1* phylogeny, we observed a unique and diversified species-specific *GCYC* expression patterns that evolved after duplication into *GCYC1C*/*1D* and *GCYC1A*/*1B* copies (Fig 6). This indicated that no specific *GCYC1* copy was exclusively correlated with the establishment and/or maintenance of either zygomorphy or actinomorphy in Trichosporeae species. Particularly for zygomorphic species, these *GCYC* copies seemed to be randomly fixed with one retaining dorsally specific expression but the other deviated to a new expression pattern. Thus, we propose that random fixation occurred at each species generating a possible “evolutionary flexibility” among *GCYC1C*/*1D* duplicates in Trichosporeae. Moreover, the PAML result suggest that those *GCYC1C*/*1D* duplicates were maintained by relaxed purifying selection. This might allow these *GCYC1* copies to co-exist or co-function throughout their divergence without immediately being selected against, providing rooms for evolutionary flexibility (Table 1, Fig 6). Thus, in contrast to other cases of *CYC2*-like gene duplications [38, 65], the fate of each *GCYC1C*/*1D* copy in Trichosporeae species is not pre-determined but randomly fixed into a species-specific pattern.

In MADS-box gene family of angiosperms, multiple gene duplications have provided evolutionary flexibility allowing each copy to be randomly fixed to maintain its original function or acquired new roles to interact with other copies or downstream genes [75, 76]. Indeed, protein interactions exist among the *CYC*/*TB1* gene subfamily of TCP family, demonstrated that combinations of different *CYC* copies may interact each other to generate novel genetic modules, such as those reported in *Arabidopsis* and tomato [77, 78]. For example, if *CYC* expression is extended to other flower parts, it might not interact with its original *CYC*/*TB1* copies but instead interacts with another partners incorporated into new modules. This may help to generate modified flower morphologies particularly in species of Asiatic Trichosporeae that exhibit a wide diversity in flower morphologies, even within a single genus such as *Oreocharis* [19] or *Petrocodon* [18].

## Supporting information

S1 Table List of accession numbers of *GCYC* from NCBI used in this study. (DOCX)

S2 Table Primer pairs used to perform RT-PCR in this study. (DOCX)

S3 Table Parameters estimate under branch-site model A of *GCYC1C* and *GCYC1D* in tribe Trichosporeae. (DOCX)

S4 Table No Type I or Type II functional divergence detected between *GCYC1C* and *GCYC1D* clades. (DOCX)

S1 Fig Alignments of protein sequences of *GCYC1C* and *GCYC1D* genes. Partial TCP domain, ECE and R domain are outlined. The black hollow square denoted the putative sub-lineage specific motif (PSLM) of *GCYC1D* genes (see Gao et al., 2008).
(TIF)

S2 Fig Using 18S rRNA PCR amplifications on DNase (RQ1) treated RNA samples for checking residual genomic contamination. As all samples amplified nothing after PCR, this indicates the successful eliminations on genomic DNA in all RNA extractions after RQ1 DNase treatment. These DNase treated RNA samples therefore are free from DNA contaminations before undergoing reverse transcription (RT) reaction.
(a) and (b), the 18S PCR results for two biological repeats of RNA extractions among various bud stages (E, M, L: early, middle and at anthesis late stage flower buds) in *C*. *ramondioides* (*Cr18S*), *H*. *bicornuta* (*Hb18S*) and *L*. *pauciflorus* (*Lp18S*). The “n” denotes non template control.
(c) and (d), the 18S PCR results for two biological repeats of RNA extractions among dissected tissues. For *C*. *ramondioides* (*Cr18S*), p1 to p5 denotes petals of *C*. *ramondioides* corresponding to that in Fig 4; sep for sepals, pe for pooled petals, st for pooled 5 stamens, and gyn for gynoecium. For *H*. *bicornuta* (*Hb18S*) and *L*. *pauciflorus* (*Lp18S*), dp denotes dorsal petals; lp: lateral petals, vp: ventral petal, sta: pooled staminodes, st: pooled stamens and gyn: gynoecium. “n” denotes non template control.
(TIF)

S3 Fig Electrophoresis results of expression pattern (RT-PCR) of *GCYC* duplicates through floral developmental stages (biological and technical repeats) of *C*. *ramondioides*, *H*. *bicornuta* and *L*. *pauciflorus*. (a), (b), (c) and (d) represent two biological repeats each with two technical repeats of *GCYC* gene expression pattern of floral bud at early (E), middle (M) and late (L) developmental stage of *C*. *ramondioides*;
(e), (f), (g) and (h) represent two biological repeats each with two technical repeats of *GCYC* gene expression pattern of floral bud at early (E), middle (M) and late (L) developmental stage of *H*. *bicornuta*;
(i), (j), (k) and (l) represent two biological repeats each with two technical repeats of *GCYC* gene expression pattern of floral bud at early (E), middle (M) and late (L) developmental stage of *L*. *pauciflorus*.
PCR products of each sample were examined at 25, 30, 35 and 40 cycles to ensure whether certain *GCYC* copy is expressed (presence of band) or not (absence of band).
(TIF)

S4 Fig Electrophoresis results of expression pattern (RT-PCR) of *GCYC* duplicates among dissected floral tissues (biological and technical repeats) of *C*. *ramondioides*, *H*. *bicornuta* and *L*. *pauciflorus*. (a), (b), (c) and (d) represent two biological repeats each with two technical repeats of *GCYC* gene expression pattern of dissected tissue of *C*. *ramondioides*;
(e), (f), (g) and (h) represent two biological repeats each with two technical repeats of *GCYC* gene expression pattern of dissected tissue of *H*. *bicornuta*;
(i), (j), (k) and (l) represent two biological repeats each with two technical repeats of *GCYC* gene expression pattern of dissected tissue of *L*. *pauciflorus*.
For *C*. *ramondioides*, p1 to p5 denotes petals of *C*. *ramondioides* corresponding to that in Fig 4; sep for sepals, pe for pooled petals, st for pooled 5 stamens, and gyn for gynoecium. For *H*. *bicornuta* and *L*. *pauciflorus*, dp denotes dorsal petals; lp: lateral petals, vp: ventral petal, sta: pooled staminodes, st: pooled stamens and gyn: gynoecium. “n” denotes non template control.
PCR products of each sample were examined at 25, 30, 35 and 40 cycles to ensure whether certain *GCYC* copy is expressed (presence of band) or not (absence of band).
(TIF)

S5 Fig Primer dimer identified from gel picture of amplification of *CrCYC1C* of *C*. *ramondioides*. P1, P2, P3, P4 and P5 corresponds to five petals of *C*. *ramondioides* in Fig 2. sep: pooled sepals of *C*. *ramondioides*. P1 to p5: pooled *C*. *ramondioides* petals; st: pooled stamens of *C*. *ramondioides*; gyn: gynoecium of *C*. *ramondioides*.
(TIF)

S1 File Alignment of all *CYC*-like genes used in this study. (FAS)

S2 File Alignment of *GCYC* duplicates used in codeml analysis. (FAS)
