# Supplementary material for: Gene duplication and relaxation from selective constraints of GCYC genes correlated with various floral symmetry patterns in Asiatic Gesneriaceae tribe Trichosporeae
Source: PLoS One. 2019 Jan 30;14(1):e0210054. doi: 10.1371/journal.pone.0210054 (PMC6353098; doi:10.1371/journal.pone.0210054)
Supplement: S1 Table — (DOCX) [file pone.0210054.s001.docx]

**S1 Table. List of accession numbers of *GCYC* from NCBI used in this study**

| **Families/**  **Subfamilies** | **Tribe** | **Subtribe** | **Species** | ***GCYC* copy** | **NCBI Accession Number** |
| --- | --- | --- | --- | --- | --- |
| **Calceolariaceae** |  |  | *Calceolaria arachnoidea* | *CaaCYC1* | AY423143 |
|  |  |  | *Calceolaria arachnoidea* | *CaaCYC2* | AY423144 |
| **Gesneriaceae/** |  |  |  |  |  |
| Gesnerioideae | Titanotricheae | - | *Titanotrichum oldhamii* | *ToCYC1* | AY423150 |
|  | Napeantheae | - | *Napeanthus sp.* | *NasCYC1* | **MG989445** |
|  |  | - | *Napeanthus reitzii* | *NarCYC1* | AY423149 |
|  | Beslerieae | Besleriinae | *Besleria labiosa* | *BelCYC1* | AY423148 |
|  | Coronanthereae | Coronantherinae | *Coronanthera clarkeana* | *CcCYC1E* | AY363952 |
|  |  |  | *Coronanthera clarkeana* | *CcCYC1F* | DQ406720 |
|  |  | Mitrariinae | *Asteranthera ovata* | *AoCYC1E* | AY363949 |
|  |  |  | *Asteranthera ovata* | *AoCYC1F* | AY363950 |
|  |  |  | *Fieldia australis* | *FaCYC1E* | AY423151 |
|  |  |  | *Fieldia australis* | *FaCYC1F* | AY423152 |
|  |  |  | *Mitraria coccinea* | *McCYC1E* | **MG989462** |
|  |  |  | *Mitraria coccinea* | *McCYC1F* | AY363953 |
|  |  | Negriinae | *Depanthus glaber* | *DGCYC1E* | DQ406723 |
|  |  |  | *Depanthus glaber* | *DGCYC1F* | DQ406727 |
|  |  |  | *Lenbrassia australiana* | *LaCYC1E* | DQ406722 |
|  |  |  | *Lenbrassia australiana* | *LaCYC1F* | DQ406726 |
|  |  |  | *Negria rhabdothamnoides* | *NrCYC1E* | **MG989463** |
|  |  |  | *Negria rhabdothamnoides* | *NrCYC1F* | **MG989464** |
|  | Gesnerieae | Gesneriinae | *Gesneria christii* | *GcCYC1* | **MG989451** |
|  |  |  | *Gesneria ventricosa* | *GvCYC1* | AY626226 |
|  |  |  | *Rhytidophyllum leucomallon* | *RlCYC1* | **MG989452** |
|  |  |  | *Rhytidophyllum tomentosum* | *RtCYC1* | AY363926 |
|  |  | Gloxiniinae | *Diastema racemiferum* | *DrCYC1* | **MG989453** |
|  |  |  | *Gloxinia perennis* | *GpCYC1* | **MG989454** |
|  |  |  | *Goyazia rupicola* | *GrCYC1* | AY363922 |
|  |  |  | *Kohleria hirsute* | *KhCYC1* | **MG989455** |
|  |  |  | *Kohleria xlucianii* | *KxCYC1* | **MG989456** |
|  |  |  | *Niphaea obolonga* | *NoCYC1* | **MG989457** |
|  |  |  | *Smithiantha multiflora* | *SmCYC1* | **MG989458** |
|  |  |  | *Smithiantha zebrina* | *SzCYC1* | **MG989459** |
|  |  | Columneinae | *Alloplectus panamensis* | *ApaCYC1* | AY363933 |
|  |  |  | *Alsobia punctate* | *ApuCYC1* | **MG989446** |
|  |  |  | *Chrysothemis pulchella* | *CpCYC1* | **MG989447** |
|  |  |  | *Columnea byrsina* | *CbCYC1* | AY363931 |
|  |  |  | *Columnea linearis* | *ClCYC1* | **MG989448** |
|  |  |  | *Episcia cupreata* | *EcCYC1* | **MG989449** |
|  |  |  | *Nautilocalyx lynchii* | *NlCYC1* | **MG989450** |
|  |  | Sphaerorrhizinae | *Spaherorrhiza sarmentiana* | *SpsCYC1* | GQ497208 |
|  |  | Ligeriinae | *Paliavana prasinata* | *PpCYC1* | **MG989460** |
|  |  |  | *Sinningia schiffneri* | *SscCYC1* | AY363941 |
|  |  |  | *Sinningia speciosa* | *SspCYC1* | AY363942 |
|  |  |  | *Sinningia* sp. | *SsCYC1* | **MG989461** |
| Didymocarpoideae | Trichosporeae | Leptoboeinae | *Rhynchotechum discolor* | *RdCYC1* | **MG989465** |
|  |  | Ramondinae | *Haberlea rhodopensis* | *HrCYC1* | **MG989466** |
|  |  |  | *Haberlea ferdinandi-coburgii* | *HfCYC1* | AF208322 |
|  |  |  | *Haberlea ferdinandi-coburgii* | *HfCYC2* | AF208317 |
|  |  |  | *Jancaea heldreichii* | *JhCYC1* | AF208332 |
|  |  |  | *Ramonda myconi* | *RmCYC1* | AF208331 |
|  |  |  | *Ramonda myconi* | *RmCYC2* | AF208318 |
|  |  | Streptocarpinae | *Streptocarpus ionanthus* | *SiCYC1A* | **MG989467** |
|  |  |  | *Streptocarpus ionanthus* | *SiCYC1B* | **MG989468** |
|  |  |  | *Streptocarpus ion.* subsp*. velutinus* | *SvCYC1A* | EF127811 |
|  |  |  | *Streptocarpus ion.* subsp*. velutinus* | *SvCYC1B* | EF127812 |
|  |  |  | *Streptocarpus dunnii* | *SdCYC1A* | AF208339 |
|  |  |  | *Streptocarpus dunnii* | *SdCYC1B* | AF208335 |
|  |  |  | *Streptocarpus primulifolius* | *SpCYC1A* | AF208340 |
|  |  |  | *Streptocarpus primulifolius* | *SpCYC1B* | AF208336 |
|  |  |  | *Streptocarpus rexii* | *SrCYC1A* | **MG989469** |
|  |  |  | *Streptocarpus rexii* | *SrCYC1B* | **MG989470** |
|  |  |  | *Streptocarpus rexii* | *SrCYC2* | **MG989471** |
|  |  | Didymocarpinae | *Aeschynanthus acuminatus* | *AaCYC1* | **MG989472** |
|  |  |  | *Aeschynanthus acuminatus* | *AaCYC2A* | **MG989473** |
|  |  |  | *Aeschynanthus acuminatus* | *AaCYC2B* | **MG989474** |
|  |  |  | *Oreocharis leiophylla* | *BlCYC1* | EF486283 |
|  |  |  | *Oreocharis leiophylla* | *BlCYC2* | EF486284 |
|  |  |  | *Conandron ramondioides* | *CrCYC1C* | **MG989475** |
|  |  |  | *Conandron ramondioides* | *CrCYC1D* | **MG989476** |
|  |  |  | *Conandron ramondioides* | *CrCYC2* | **MG989477** |
|  |  |  | *Cyrtandra apiculata* | *CaCYC1* | AY423160 |
|  |  |  | *Cyrtandra apiculata* | *CaCYC2* | AY423147 |
|  |  |  | *Didymocarpus citrinus* | *DcCYC1C* | AY423158 |
|  |  |  | *Didymocarpus citrinus* | *DcCYC1D* | AY423162 |
|  |  |  | *Hemiboea bicornuta* | *HbCYC1C* | **MG989478** |
|  |  |  | *Hemiboea bicornuta* | *HbCYC1D* | **MG989479** |
|  |  |  | *Hemiboea bicornuta* | *HbCYC2A* | **MG989480** |
|  |  |  | *Hemiboea bicornuta* | *HbCYC2B* | **MG989481** |
|  |  |  | *Loxostigma sp.* | *LsCYC1C* | AY423161 |
|  |  |  | *Loxostigma sp.* | *LsCYC1D* | AY423162 |
|  |  |  | *Lysionotus pauciflorus* | *LpCYC1C* | **MG989482** |
|  |  |  | *Lysionotus pauciflorus* | *LpCYC1D* | **MG989483** |
|  |  |  | *Lysionotus pauciflorus* | *LpCYC2A* | **MG989484** |
|  |  |  | *Lysionotus pauciflorus* | *LpCYC2B* | **MG989485** |
|  |  |  | *Oreocharis dinghushanensis* | *OdCYC1C* | FJ710518 |
|  |  |  | *Oreocharis dinghushanensis* | *OdCYC1D* | FJ710519 |
|  |  |  | *Oreocharis dinghushanensis* | *OdCYC2A* | FJ710520 |
|  |  |  | *Oreocharis dinghushanensis* | *OdCYC2B* | FJ644637 |
|  |  |  | *Oreocharis benthamii* | *ObCYC1* | FJ710517 |
|  |  |  | *Oreocharis benthamii* | *ObCYC2* | FJ710516 |
|  |  |  | *Paraboea swinhoei* | *PsCYC1* | **MG989486** |
|  |  |  | *Paraboea swinhoei* | *PsCYC2* | **MG989487** |
|  |  |  | *Primulina heterotricha* | *PhCYC1C* | JX020500 |
|  |  |  | *Primulina heterotricha* | *PhCYC1D* | JX020501 |
|  |  |  | *Primulina heterotricha* | *PhCYC2A* | JX020502 |
|  |  |  | *Primulina heterotricha* | *PhCYC2B* | JX020503 |
|  |  |  | *Primulina tabacum* | *PtCYC1* | AF208328 |
|  |  |  | *Primulina tabacum* | *PtCYC2* | **MG989488** |
|  | **Epithemateae** |  | *Epithema benthamii* | *EbCYC1* | AY423157 |
|  |  |  | *Epithema benthamii* | *EbCYC2* | AY423146 |

Accession numbers in bold were sequenced in this study.
