# Supplementary material for: Gene duplication and relaxation from selective constraints of GCYC genes correlated with various floral symmetry patterns in Asiatic Gesneriaceae tribe Trichosporeae
Source: PLoS One. 2019 Jan 30;14(1):e0210054. doi: 10.1371/journal.pone.0210054 (PMC6353098; doi:10.1371/journal.pone.0210054)
Supplement: S2 Table — (DOCX) [file pone.0210054.s002.docx]

**S2 Table. Primer pairs used to perform RT-PCR in this study**

| *CrCYC1C* |  |
| --- | --- |
| Forward | 5'-AGACATGCTTTCTGGCCACT-3' |
| Reverse | 5'-CTTCTTCGCCTTCTGAATGC-3' |
| *CrCYC1D* |  |
| Forward | 5’-CAGGTGCAGATTCGATGAGA-3’ |
| Reverse | 5’-GTTCCATTGCAGTCTCCCAT-3’ |
| *CrCYC2* |  |
| Forward | 5’-TCTTGCTTCATCAGCACCAC-3’ |
| Reverse | 5’-GTGATGCCCCTACTTGCACT-3’ |
| *HbCYC1C* |  |
| Forward | 5'-AGAGCAAGGGCTAGGGAAAG-3' |
| Reverse | 5'-CCCAATTCGCATTGACATTA-3' |
| *HbCYC1D* |  |
| Forward | 5'-AGGTTTCGACAAGCCAAAGA-3' |
| Reverse | 5'-TGGTCTGCAAACTTCAAACG-3' |
| *HbCYC2A* |  |
| Forward | 5'-GGGAACTTTGTCCAAGCAAA-3' |
| Reverse | 5'-GCTGAAACCCGAAAATTGAA-3' |
| *HbCYC2B* |  |
| Forward | 5'-AGAGCAAGGGCTAGGGAAAG-3' |
| Reverse | 5'-GTTGGATTGTGCTGTTGAGC-3' |
| *LpCYC1C* |  |
| Forward | 5'-AGKTGTTTATTACCTGCGGA-3' |
| Reverse | 5'-TTGTGCTGATCCAAAATGTCA-3' |
| *LpCYC1D* |  |
| Forward | 5'-GGAGCACTTCTTCCCCTTCT-3' |
| Reverse | 5'-ATGGGAGACTGCAATGGAAC-3' |
| *LpCYC2A* |  |
| Forward | 5'-TGGCAAATACTTACAGATGTAT-3' |
| Reverse | 5'-TTGTGCTGATCCAAAATGTCA-3' |
| *LpCYC2B* |  |
| Forward | 5'-ACGAAATCGAAAGCAGCAAT-3' |
| Reverse | 5'-GAAAATGGAGGCTAGGCACA-3' |
| 18S |  |
| Forward | 5’-CTTCGGGATCGGAGTAATGA-3’ |
| Reverse | 5’-GGTAAGTTTCCCCGTGTTGA-3’ |
