# Supplementary material for: Gene duplication and relaxation from selective constraints of GCYC genes correlated with various floral symmetry patterns in Asiatic Gesneriaceae tribe Trichosporeae
Source: PLoS One. 2019 Jan 30;14(1):e0210054. doi: 10.1371/journal.pone.0210054 (PMC6353098; doi:10.1371/journal.pone.0210054)
Supplement: S3 Table — (DOCX) [file pone.0210054.s003.docx]

**S3 Table. Parameters estimate under branch-site model A of *GCYC1C* and *GCYC1D* in tribe Trichosporeae**
