# Supplementary material for: Gene duplication and relaxation from selective constraints of GCYC genes correlated with various floral symmetry patterns in Asiatic Gesneriaceae tribe Trichosporeae
Source: PLoS One. 2019 Jan 30;14(1):e0210054. doi: 10.1371/journal.pone.0210054 (PMC6353098; doi:10.1371/journal.pone.0210054)
Supplement: S4 Table — (DOCX) [file pone.0210054.s004.docx]

**S4 Table. No Type I or Type II functional divergence detected between *GCYC1C* and *GCYC1D* clades.**

|  | **Type I** |  |  |  |
| --- | --- | --- | --- | --- |
|  | θML | θSE | LRTθ | *P*-value |
| ***GCYC1C*/*GCYC1D*** | na | na | na | - |
|  | **Type II** |  |  |  |
|  | AlphaML | θ_II_ | θ_II_ SE | aR/πR |
| ***GCYC1C*/*GCYC1D*** | -7.83119 | -0.0907 | 0.041452 | 1.16129 |
