# Supplementary figures and images for: Gene duplication and relaxation from selective constraints of GCYC genes correlated with various floral symmetry patterns in Asiatic Gesneriaceae tribe Trichosporeae
Source: PLoS One. 2019 Jan 30;14(1):e0210054. doi: 10.1371/journal.pone.0210054 (PMC6353098; doi:10.1371/journal.pone.0210054)

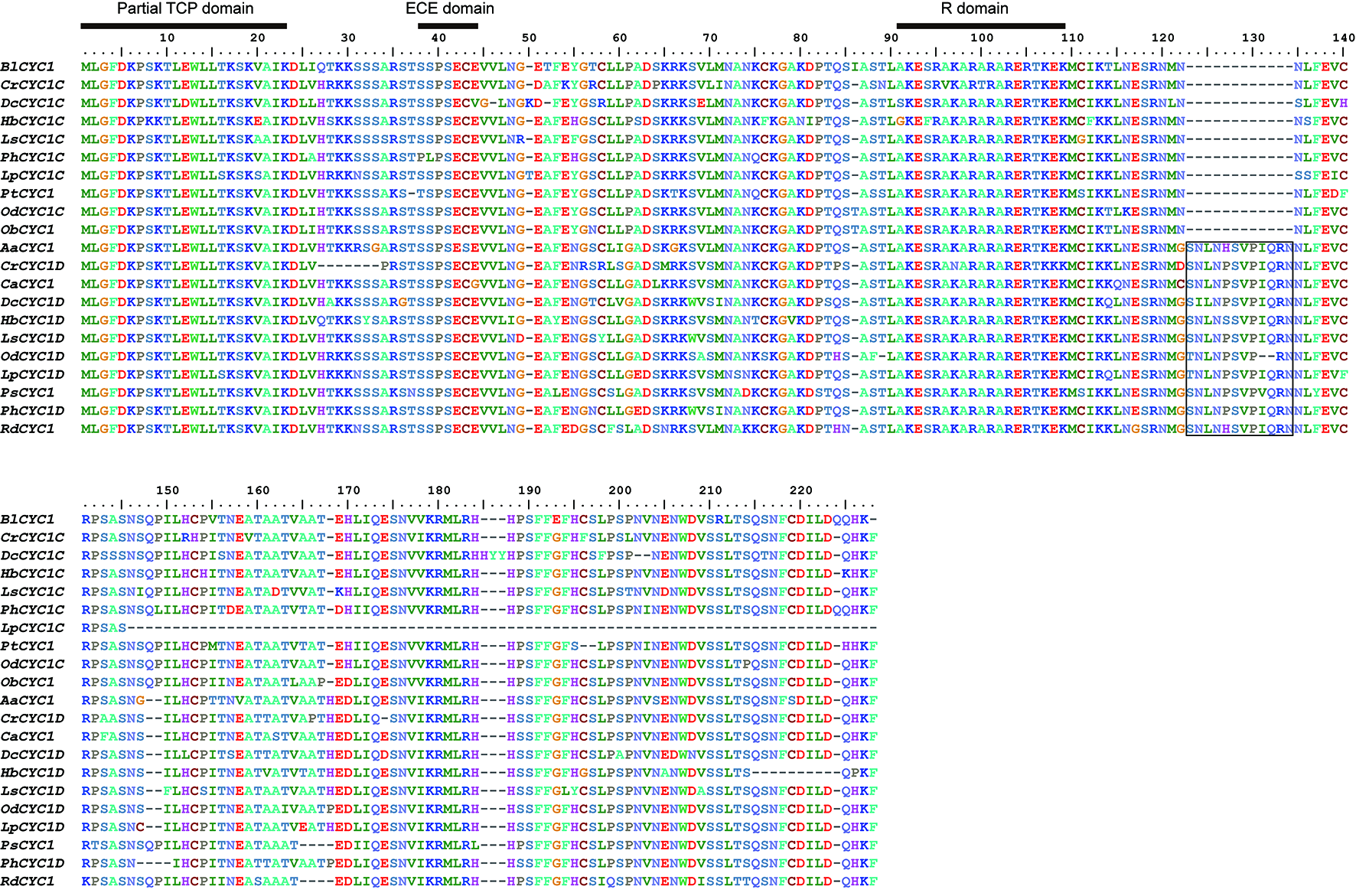

Supplement: S1 Fig — Partial TCP domain, ECE and R domain are outlined. The black hollow square denoted the putative sub-lineage specific motif (PSLM) of GCYC1D genes (see Gao et al., 2008). (TIF) [file pone.0210054.s005.tif]

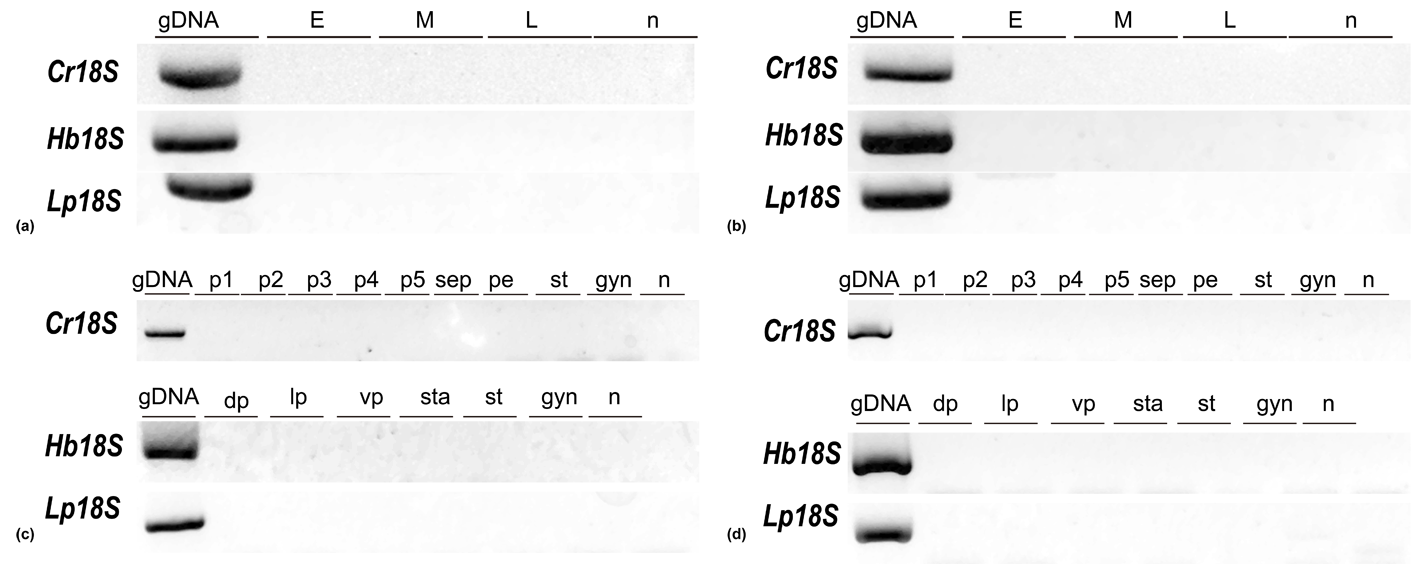

Supplement: S2 Fig — As all samples amplified nothing after PCR, this indicates the successful eliminations on genomic DNA in all RNA extractions after RQ1 DNase treatment. These DNase treated RNA samples therefore are free from DNA contaminations before undergoing reverse transcription (RT) reaction. (a) and (b), the 18S PCR results for two biological repeats of RNA extractions among various bud stages (E, M, L: early, middle and at anthesis late stage flower buds) in C. ramondioides (Cr18S), H. bicornuta (Hb18S) and L. pauciflorus (Lp18S). The “n” denotes non template control. (c) and (d), the 18S PCR results for two biological repeats of RNA extractions among dissected tissues. For C. ramondioides (Cr18S), p1 to p5 denotes petals of C. ramondioides corresponding to that in Fig 4; sep for sepals, pe for pooled petals, st for pooled 5 stamens, and gyn for gynoecium. For H. bicornuta (Hb18S) and L. pauciflorus (Lp18S), dp denotes dorsal petals; lp: lateral petals, vp: ventral petal, sta: pooled staminodes, st: pooled stamens and gyn: gynoecium. “n” denotes non template control. (TIF) [file pone.0210054.s006.tif]

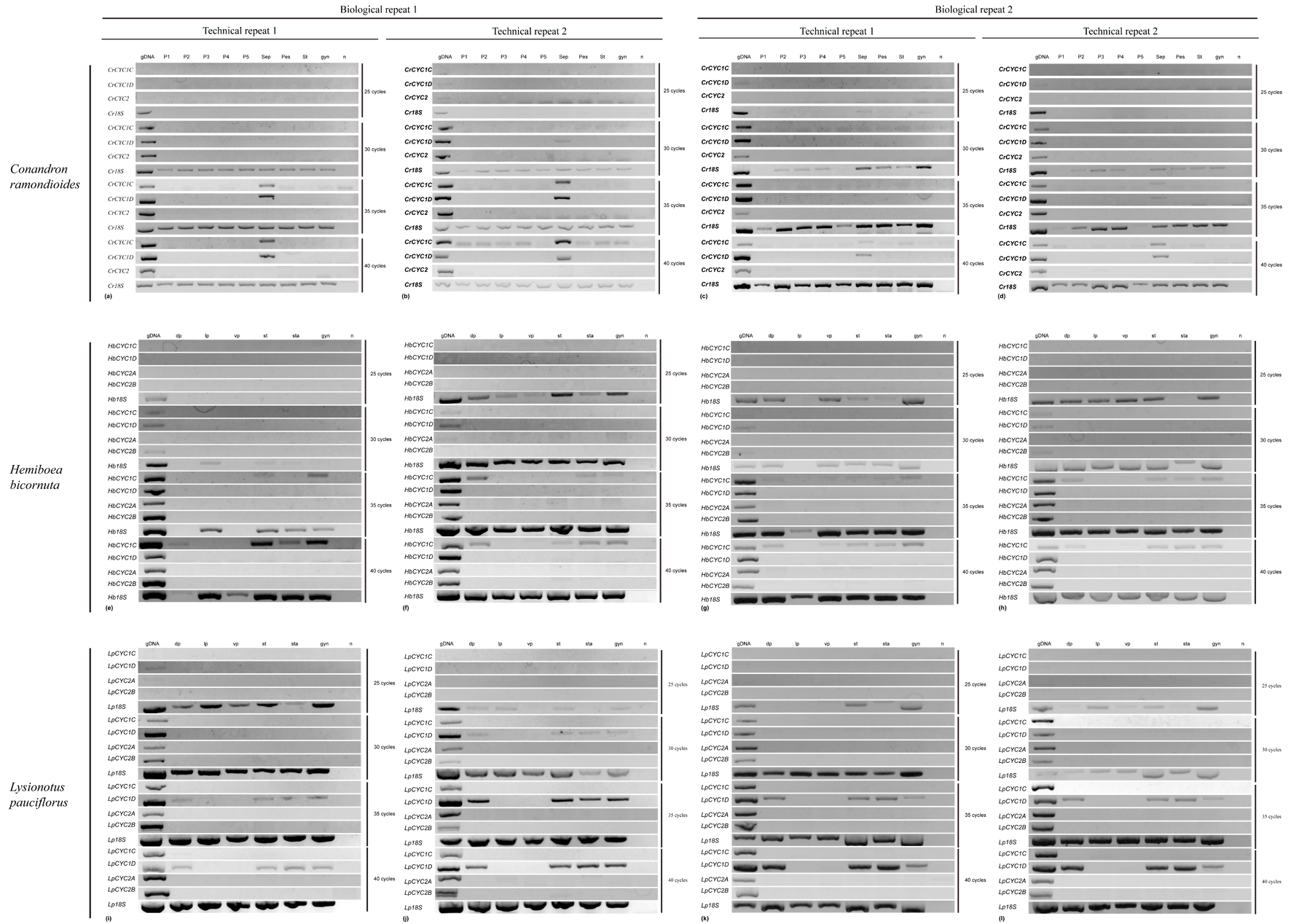

Supplement: S4 Fig — (a), (b), (c) and (d) represent two biological repeats each with two technical repeats of GCYC gene expression pattern of dissected tissue of C. ramondioides; (e), (f), (g) and (h) represent two biological repeats each with two technical repeats of GCYC gene expression pattern of dissected tissue of H. bicornuta; (i), (j), (k) and (l) represent two biological repeats each with two technical repeats of GCYC gene expression pattern of dissected tissue of L. pauciflorus. For C. ramondioides, p1 to p5 denotes petals of C. ramondioides corresponding to that in Fig 4; sep for sepals, pe for pooled petals, st for pooled 5 stamens, and gyn for gynoecium. For H. bicornuta and L. pauciflorus, dp denotes dorsal petals; lp: lateral petals, vp: ventral petal, sta: pooled staminodes, st: pooled stamens and gyn: gynoecium. “n” denotes non template control. PCR products of each sample were examined at 25, 30, 35 and 40 cycles to ensure whether certain GCYC copy is expressed (presence of band) or not (absence of band). (TIF) [file pone.0210054.s008.tif]

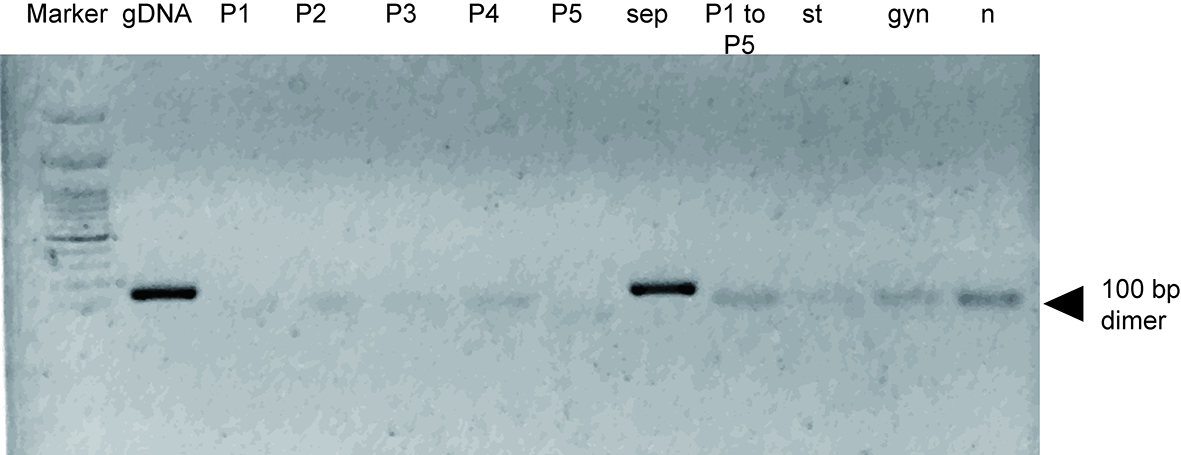

Supplement: S5 Fig — P1, P2, P3, P4 and P5 corresponds to five petals of C. ramondioides in Fig 2. sep: pooled sepals of C. ramondioides. P1 to p5: pooled C. ramondioides petals; st: pooled stamens of C. ramondioides; gyn: gynoecium of C. ramondioides. (TIF) [file pone.0210054.s009.tif]
